# Adjustment of Photosynthetic and Antioxidant Activities to Water Deficit Is Crucial in the Drought Tolerance of *Lolium multiflorum/Festuca arundinacea* Introgression Forms

**DOI:** 10.3390/ijms21165639

**Published:** 2020-08-06

**Authors:** Katarzyna Lechowicz, Izabela Pawłowicz, Dawid Perlikowski, Magdalena Arasimowicz-Jelonek, Sara Blicharz, Aleksandra Skirycz, Adam Augustyniak, Robert Malinowski, Marcin Rapacz, Arkadiusz Kosmala

**Affiliations:** 1 Institute of Plant Genetics, Polish Academy of Sciences, Strzeszyńska 34, 60-479 Poznań, Poland; kmas@igr.poznan.pl (K.L.); dper@igr.poznan.pl (D.P.); sbli@igr.poznan.pl (S.B.); aaug@igr.poznan.pl (A.A.); rmal@igr.poznan.pl (R.M.); akos@igr.poznan.pl (A.K.); 2 Department of Plant Ecophysiology, Institute of Experimental Biology, Faculty of Biology, Adam Mickiewicz University, Uniwersytetu Poznańskiego 6, 61-614 Poznań, Poland; arasim@amu.edu.pl; 3 Department of Molecular Physiology, Max-Planck Institute of Molecular Plant Physiology, Am Mühlenberg 1, 14476 Potsdam-Golm, Germany; skirycz@mpimp-golm.mpg.de; 4 Department of Plant Breeding, Physiology and Seed Science, University of Agriculture in Kraków, Podłużna 3, 30-239 Kraków, Poland; rrrapacz@cyf-kr.edu.pl

**Keywords:** antioxidant enzymes, drought, forage grasses, *Lolium multiflorum/Festuca arundinacea*, stomata, reactive oxygen species, photosynthesis, triacylglycerol lipids

## Abstract

*Lolium multiflorum/Festuca arundinacea* introgression forms have been proved several times to be good models to identify key components of grass metabolism involved in the mechanisms of tolerance to water deficit. Here, for the first time, a relationship between photosynthetic and antioxidant capacities with respect to drought tolerance of these forms was analyzed in detail. Two closely related *L. multiflorum/F. arundinacea* introgression forms distinct in their ability to re-grow after cessation of prolonged water deficit in the field were selected and subjected to short-term drought in pots to dissect precisely mechanisms of drought tolerance in this group of plants. The studies revealed that the form with higher drought tolerance was characterized by earlier and higher accumulation of abscisic acid, more stable cellular membranes, and more balanced reactive oxygen species metabolism associated with a higher capacity of the antioxidant system under drought conditions. On the other hand, both introgression forms revealed the same levels of stomatal conductance, CO_2_ assimilation, and consequently, intrinsic water use efficiency under drought and recovery conditions. However, simultaneous higher adjustment of the Calvin cycle to water deficit and reduced CO_2_ availability, with respect to the accumulation and activity of plastid fructose-1,6-bisphosphate aldolase, were clearly visible in the form with higher drought tolerance.

## 1. Introduction

Drought events that occur in Europe more frequently nowadays [1] are among the most harmful environmental factors affecting plant productivity [2]. Water shortage reduces photosynthetic metabolism and this phenomenon could result in alterations of water use efficiency (WUE) [3]. Furthermore, a requirement of water saving under drought conditions causes a significant reduction of gas exchange that leads to a decrease in carbon assimilation and consequently, to an excess of absorbed light energy. These processes in turn can trigger overproduction of reactive oxygen species (ROS) [4]. A reduction of photosynthetic efficiency can be due to stomatal (L_S_) and non-stomatal limitations (L_NS_), also called biochemical limitations. L_NS_ cover mesophyll conductance, carbon metabolism with the Calvin cycle [5,6,7], source–sink dynamics, and leaf ultrastructure [8,9,10]. The adjustment in stomatal conductance is a key factor in WUE optimization under unfavorable conditions and it is controlled also by stomatal density (SD) and stomatal size (SS) [2,11,12,13,14,15]. In the epidermis of grasses, a stomatal complex is found which is constructed of dumbbell-shaped guard cells (GC) and neighboring subsidiary cells (SC) [16,17,18,19]. Many observations suggest that physical interactions between guard cells and subsidiary cells in monocots trigger faster and more effective stomatal movement than in dicots [20,21,22]. Moreover, subsidiary cells are crucial in supplying ions to guard cells, which evokes changes in turgor pressure that control stomatal aperture [23,24]. However, SS and SD can also be modified by long-term external stimuli, including drought, to optimize stomatal conductance during water saving [2,25].

Under drought conditions, cellular processes involved in photoprotection play fundamental roles in plant metabolism. These processes involve the activity of the antioxidant system, dissipation of absorbed light energy as a heat, the photorespiration pathway [26], and utilization of excess light energy for the production of reduced-carbon compounds, including storage lipids [27]. A relationship between drought tolerance and the abundance and activity of antioxidant enzymes has been observed in different plant species [4,28]. It has been demonstrated that abscisic acid (ABA)-inducible accumulation of triacylglycerol lipids (TAG) in the vegetative tissues represents a common mechanism of plant adaptation to different abiotic stresses, including long-term stress acclimation [29,30]. In plants, TAG accumulation has been observed under heat stress [31], drought [29,32,33], prolonged darkness [34], and excess light [27]. It was demonstrated that the accumulation of TAG can protect cells against oxidative stress by limiting ROS generation and inhibiting lipid peroxidation of polyunsaturated fatty acids [30]. The concentration of free fatty acids (FA), which are highly toxic for the cell environment under stress conditions, inducing ROS production, is highly regulated by feedback inhibition of FA biosynthesis by the oleic acid-acyl carrier protein and by the sequestration of excess free FA into triacylglycerol lipids, which are stored in lipid droplets [35].

Grasslands comprise complex and economically important ecosystems worldwide. In temperate regions, including Europe, two genera of forage grasses, *Lolium* and *Festuca*, are widely distributed. *Lolium* species are characterized by good pasture and forage quality, while *Festuca* species have excellent tolerance to abiotic stresses, including water deficit [36,37,38,39,40]. Although *L. multiflorum* (Italian ryegrass) can express some levels of drought tolerance, its yield can be dramatically reduced under water deficiency [41,42,43]. Otherwise, *F. arundinacea* (tall fescue) has excellent potential to survive water deficit, based on strategies of avoidance, tolerance, and recovery [33,44,45]. *L. multiflorum/F. arundinacea* introgression forms were previously applied as models in studies on drought tolerance, including analysis of the leaf proteome, primary metabolome, and lipidome [32,46,47], as well as the architecture and metabolism of roots, including the analysis of the primary metabolome and lipidome [40]. To date, metabolic networks associated with photosynthetic and antioxidant capacities under drought have not been fully understood in forage grasses. Thus, we subjected, for the first time, *L. multiflorum/F. arundinacea* introgression forms distinct in their levels of drought tolerance to comprehensive research to decipher the relationship between photosynthesis and enzymatic antioxidant activity under drought and recovery conditions in forage grasses. Moreover, we assumed that the adjustment of the Calvin cycle to the conditions of water deficit could be one of the most crucial traits in *Lolium–Festuca* grasses to survive drought. The study presented in this paper involves: (*i*) analysis of the plant physiological performance, including ABA and TAG lipid accumulation, electrolyte leakage (EL), lipid peroxidation, gas exchange (CO_2_ assimilation (A), stomatal conductance (g_s_), transpiration (E), internal CO_2_ concentration (*Ci*)), and the relative water content (RWC) in leaf tissue; (*ii*) analysis of the photosynthetic capacity (chlorophyll fluorescence; gene expression, protein accumulation, and activity of two Calvin cycle enzymes: plastid fructose-1,6-bisphosphate aldolase (pFBA, EC 4.1.2.13) and plastid phosphoglycerate kinase (pPGK, EC 2.7.2.3)); (*iii*) analysis of the antioxidant capacity (ROS generation; gene expression, protein accumulation and activity of antioxidant enzymes: glutathione reductase (GR, EC 1.6.4.2), glutathione peroxidase (GPX, EC 1.11.1.9), L-ascorbate peroxidase (APX, EC 1.11.1.11), catalase (CAT, EC 1.11.1.6), and superoxide dismutases (SOD, EC 1.15.1.1): iron superoxide dismutase (Fe-SOD), copper/zinc superoxide dismutase (Cu/Zn-SOD), and manganese superoxide dismutase (Mn-SOD)). The structure and density of stomatal complexes were also recognized.

## 2. Results

### 2.1. Selection of L. multiflorum/F. arundinacea Introgression Forms with Distinct Levels of Drought Tolerance in the Field Conditions

The meteorological conditions between May and September 2014 are presented in Figure S1D. Re-growth (Figure S1B) and bonitation (Figure S1C) measurements demonstrated that under prolonged drought, the yield of the whole population of the *L. multiflorum/F. arundinacea* introgression forms was progressively reduced. A range of diversity with respect to these traits among individual forms was also observed. However, after re-watering, not all the introgression forms resumed their growth; thus, we decided that the ability to re-grow after stress cessation could be a good trait to select plants for further research. The scores for the high-drought-tolerant (HDT) form, which was able to re-grow after re-watering, and for the low-drought-tolerant (LDT) form, which did not survive the drought period, as well as the mean scores for the whole population, are presented in Figure S1B,C. The HDT and the LDT introgression forms did not differ with respect to the analyzed parameters under optimal (installation) and water deficit conditions.

### 2.2. Physiological Performance of the LDT and HDT Introgression Forms under Drought and Re-Watering Conditions in the Environmental Chamber

**Figure 1:**
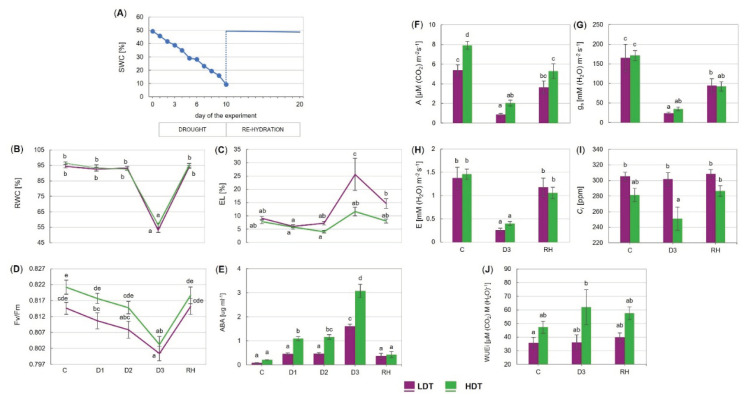
(**A**) Changes in soil water capacity (SWC) in the pots during the experiment. (**B**) Relative water content (RWC), (**C**) electrolyte leakage (EL), (**D**) maximum quantum efficiency of photosystem II photochemistry (Fv/Fm), (**E**) accumulation of abscisic acid (ABA), (**F**) CO_2_ assimilation (A), (**G**) stomatal conductance (g_s_), (**H**) transpiration (E), (**I**) internal CO_2_ concentration (*Ci*), and (**J**) intrinsic water use efficiency (WUEi) of two *L. multiflorum/F. arundinacea* introgression forms (low- and high-drought tolerant—LDT and HDT, respectively) at different time points: before stress treatment (C), on the 3rd (D1), 6th (D2), and 10th (D3) day of the water deficit and 10 days after re-hydration (RH). Error bars represent the standard errors. Homogeneous groups are denoted by the same letter according to Fisher’s LSD test (*p* = 0.01).

Under severe drought, the leaves of both introgression forms presented visual symptoms of the loss of turgidity (Figure S2). After re-hydration, the leaves returned to their normal shape in both forms; nevertheless, numerous dried leaves could also be observed. The amount of water in the pots decreased gradually from the control value of around 50% of soil water capacity (SWC), reaching approximately 9% of SWC after 10 days of drought (Figure 1A). The relative water content decreased to around 50% of its maximal value in both the LDT and HDT introgression forms in the advanced stage of drought (between 6th day (D2) and 10th day (D3)) (Figure 1B), whereas electrolyte leakage increased only in the LDT form, achieving a level approximately threefold higher than that under the control conditions (Figure 1C). Both parameters resumed to the initial levels after re-watering (Figure 1B,C). A significant reduction of the maximum quantum efficiency of photosystem II (PSII) photochemistry (Fv/Fm) was visible in both introgression forms on the 10th day of stress duration but it returned to the control values after stress cessation (Figure 1D). Drought- and recovery-evoked changes in other chlorophyll fluorescence parameters in the two *L. multiflorum/F. arundinacea* introgression forms are presented in Figure S3. Gas exchange parameters were similar for both forms in the control, except CO_2_ assimilation, which was lower in the LDT introgression form (Figure 1F). During stress application, A, g_s_, and E were reduced in both introgression forms, but not the *Ci* parameter, which was stable in both forms compared with the control conditions (Figure 1F–I). After re-watering, the E parameter returned to the control values in the two analyzed introgression forms (Figure 1H). On the other hand, g_s_ remained reduced in both forms compared with the control conditions (Figure 1G). The value of A remained at a lower level compared with the initial value, only in the HDT. The intrinsic water use efficiency (WUEi) did not change significantly under drought (D3) as well as after re-watering in both introgression forms (Figure 1J). Drought-induced accumulation of ABA was observed in both introgression forms (Figure 1E). In the HDT introgression form, ABA started to increase in the initial phase of stress (D1) and reached the highest value at D3. In the LDT form, the ABA content was elevated under advanced drought (D3); however, it remained lower than that in the HDT form. After re-hydration, the ABA content decreased to the control levels in both introgression forms.

### 2.3. Stomatal Characteristics

**Figure 2:**
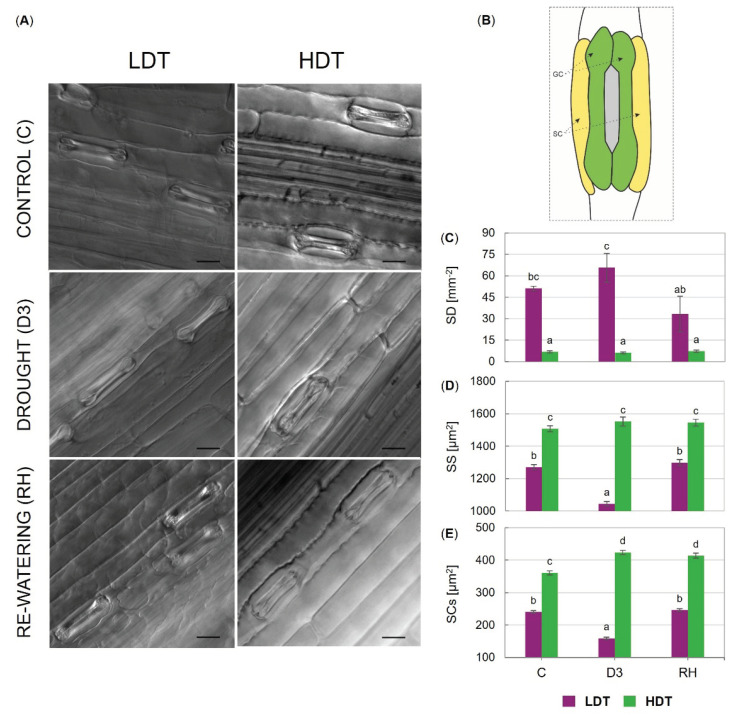
(**A**) Light micrographs illustrating leaf surfaces and stomata of two *L. multiflorum/F. arundinacea* introgression forms (low- and high-drought tolerant—LDT and HDT, respectively) before stress treatment (C), on the 10th (D3) day of the water deficit, and 10 days after re-watering (RH), scale bars 20 µm. (**B**) Schematic of the dumbbell-shaped guard cells (GC) and flanking subsidiary cells (SC) surrounding the stomatal pore. (**C**) Stomatal density (SD), (**D**) stomatal size (SS), and (**E**) subsidiary cells size (SCs) in the LDT and HDT forms at C, D3, and RH. Error bars represent the standard errors. Homogeneous groups are denoted by the same letter according to Fisher’s LSD test (*p* = 0.01).

The two tested introgression forms differed statistically from each other, where the LDT one had higher stomatal density than the HDT introgression form. However, only slight changes in stomatal density were observed during the experiment in the case of the LDT form (Figure 2C). Stomatal size and subsidiary cells size (SCs) were significantly higher in the HDT compared to the LDT form (Figure 2D,E). Furthermore, the size of stomata and subsidiary cells decreased under advanced drought in the LDT form. On the other hand, the subsidiary cells size increased slightly but significantly at D3 and RH time points compared to the control conditions in the HDT introgression form (Figure 2E). Light micrographs illustrating leaf surfaces and stomata in both introgression forms and a schematic of the dumbbell-shaped guard cells and flanking subsidiary cells surrounding the stomatal pore are presented in Figure 2A,B.

### 2.4. Expression of Genes Encoding the Calvin Cycle Enzymes at Transcript and Protein Levels

**Figure 3:**
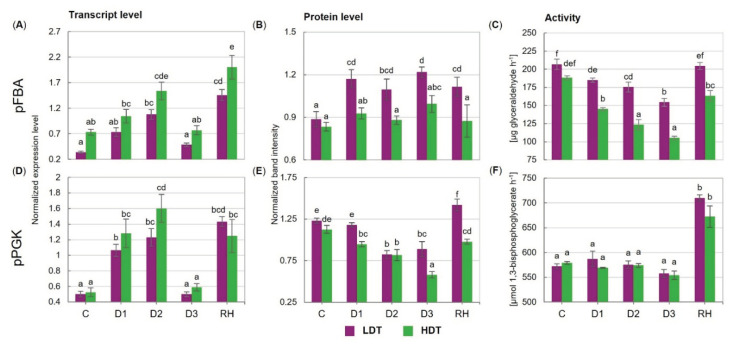
(**A**–**C**) Expression and activity of plastid fructose-1,6-bisphosphate aldolase (pFBA) and (**D**–**F**) plastid phosphoglycerate kinase (pPGK) in two *L. multiflorum/F. arundinacea* introgression forms (low- and high-drought tolerant—LDT and HDT, respectively) before stress treatment (C), on the 3rd (D1), 6th (D2), and 10th (D3) day of water deficit and 10 days after re-hydration (RH). The transcript accumulation levels of actin and ubiquitin were used as references. Error bars represent the standard errors. Homogeneous groups are denoted by the same letter according to Fisher’s LSD test (*p* = 0.01).

The transcript accumulation patterns of pFBA and pPGK were similar in both introgression forms (Figure 3A,D). These profiles were characterized by up-regulation of gene expression in the initial stage of stress treatment (between D1 and D2 time points) and its reduction to the control levels under severe drought (D3). Moreover, a significant increase in both transcript accumulation levels after stress cessation was observed in the LDT and HDT introgression forms (Figure 3A,D). Protein accumulation of pFBA was relatively stable in the HDT form during the whole experiment, while higher accumulation occurred both in the drought period and after re-watering in the LDT form (Figure 3B). Under water withholding, the protein accumulation of pPGK decreased in both introgression forms (Figure 3E). This decline was observed at D1 in the HDT form and at the D2 time point in the LDT form. The level of the pPGK protein was significantly higher in the LDT form than in the HDT form under the severe water deficit (D3). After stress cessation, the pPGK protein was highly accumulated in both introgression forms, and in the LDT form, an even higher accumulation level occurred compared with that observed under the control conditions.

### 2.5. Activities of the Calvin Cycle Enzymes

The activity of pFBA under the control conditions was similar for both introgression forms (Figure 3C). The water deficit evoked its progressive reduction, which was significantly faster in the HDT form. This phenomenon resulted in a higher activity of pFBA in the LDT compared to the HDT form during the whole drought period and after re-watering. Furthermore, after stress cessation, pFBA activity was restored to the control level only in the LDT introgression form. The activity of pPGK was not affected by drought in both introgression forms; however, it significantly increased after re-watering (Figure 3F). No differences between the analyzed introgression forms with respect to pPGK activity were revealed.

### 2.6. Expression of Genes Encoding Antioxidant Enzymes at Transcript and Protein Levels

**Figure 4:**
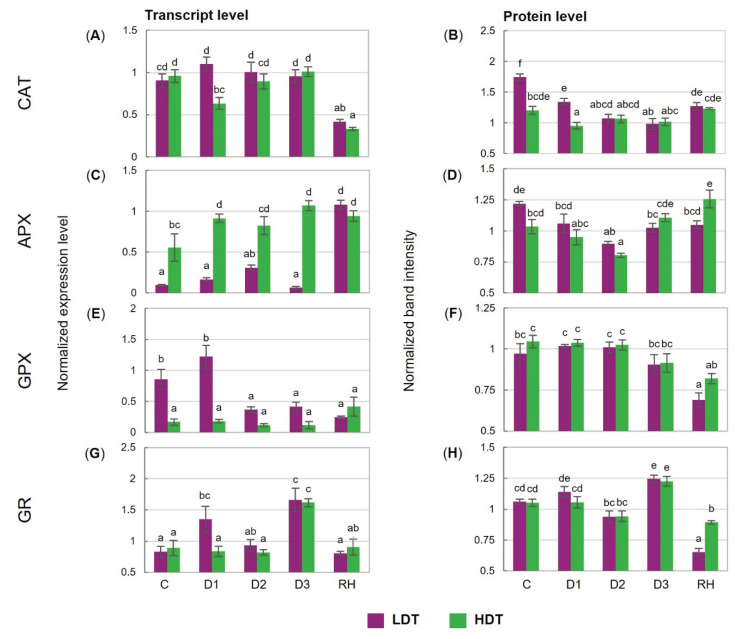

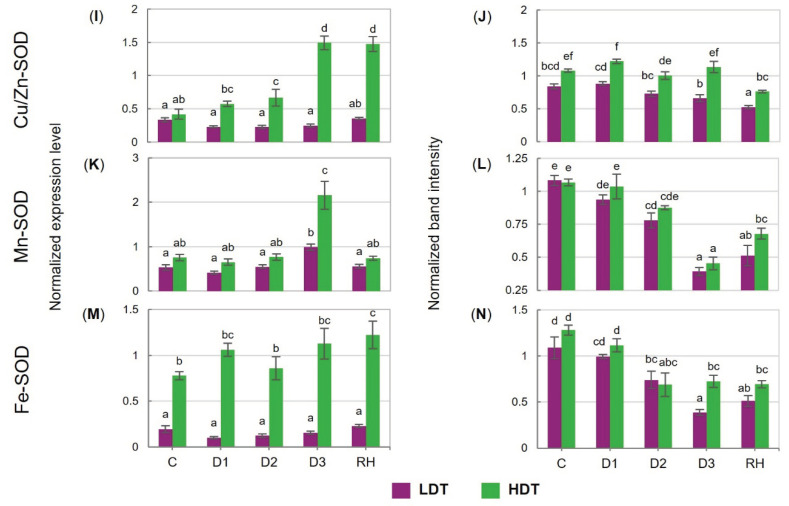
(**A**,**B**) Transcript and protein levels of catalase (CAT), (**C**,**D**) L-ascorbate peroxidase (APX), (**E**,**F**) glutathione peroxidase (GPX), (**G**,**H**) glutathione reductase (GR), (**I**,**J**) copper/zinc superoxide dismutase (Cu/Zn-SOD), (**K**,**L**) manganese superoxide dismutase (Mn-SOD), and (**M**,**N**) iron superoxide dismutase (Fe-SOD) in two *L. multiflorum/F. arundinacea* introgression forms (low- and high-drought tolerant—LDT and HDT, respectively) before stress treatment (C), on the 3rd (D1), 6th (D2), and 10th (D3) day of water deficit and 10 days after re-hydration (RH). The transcript accumulation levels of actin and ubiquitin were used as references. Error bars represent the standard errors. Homogeneous groups are denoted by the same letter according to Fisher’s LSD test (*p* = 0.01).

Under drought, the transcript accumulation of CAT was unchanged in the LDT form. Moreover, it was reduced twofold in both introgression forms after re-watering (Figure 4A). The content of the CAT protein was almost not affected by water deficiency in the HDT, while it was progressively reduced until the 6th day of stress (D2) in the LDT introgression form (Figure 4B). Different dynamics of APX transcript accumulation was observed in the two analyzed forms. It increased after stress initiation and remained stable during water deficit conditions, and also after re-watering in the HDT form. On the other hand, the LDT form increased its level only after re-hydration. Thus, the abundance of the APX transcript was remarkably higher in the HDT introgression form under both control and stress conditions (Figure 4C). In both introgression forms, the APX protein level did not differ significantly under the control and drought conditions. After re-hydration, the APX content was similar to that observed under the control conditions in the case of the LDT form or was even higher in the HDT form (Figure 4D). The expression pattern of GPX did not change during the whole experiment in the HDT form. By contrast, in the LDT form, a reduction in GPX transcript accumulation was observed at the D2 and D3 time points and it was not recovered after re-hydration (Figure 4E). Accumulation of the GPX protein was stable during the water deficit in both introgression forms but decreased significantly in the LDT form after stress cessation (Figure 4F). Transcript and protein accumulation of GR increased under advanced stress conditions and returned to lower levels after re-watering in both introgression forms (Figure 4G,H). In the LDT form, drought treatment slightly up-regulated Mn-SOD expression at D3, whereas it did not change Cu/Zn-SOD and Fe-SOD transcript accumulation (Figure 4I,K,M). In the HDT form, higher accumulation of Mn-SOD and Cu/Zn-SOD transcripts was observed under water deficit. Furthermore, under advanced drought, higher transcript accumulation of all SOD isoforms was noticed in the HDT introgression form compared with the LDT form. In both introgression forms, accumulation of the Cu/Zn-SOD protein did not reveal any significant changes under drought (Figure 4J). However, a higher content of this protein was observed in the HDT introgression form during the whole experiment. Reduced Fe-SOD and Mn-SOD protein accumulation at D3 was noticed in both introgression forms, and these lower levels, compared with the control conditions, were maintained after re-hydration; only a slight increase in the case of Mn-SOD in the HDT form was observed (Figure 4L,N).

### 2.7. Activities of Antioxidant Enzymes

**Figure 5:**
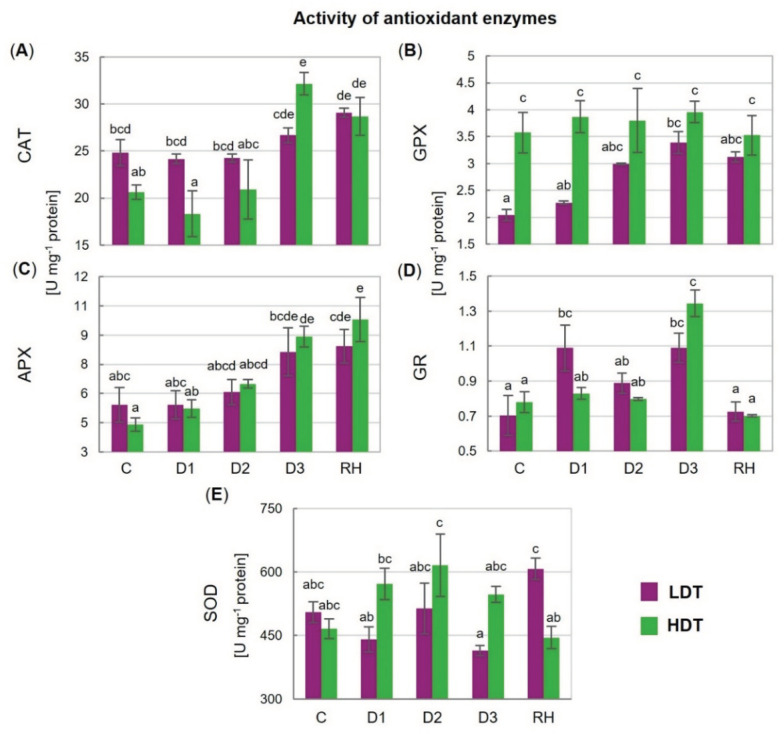
(**A**) Activity of catalase (CAT), (**B**) glutathione peroxidase (GPX), (**C**) L-ascorbate peroxidase (APX), (**D**) glutathione reductase (GR), and (**E**) superoxide dismutase (SOD) in two *L. multiflorum/F. arundinacea* introgression forms (low- and high-drought tolerant—LDT and HDT, respectively) before stress treatment (C), on the 3rd (D1), 6th (D2), and 10th (D3) day of water deficit and 10 days after re-hydration (RH). Error bars represent the standard errors. Homogeneous groups are denoted by the same letter according to Fisher’s LSD test (*p* = 0.01).

The activity of CAT increased on the 10th (D3) day of stress but only in the HDT introgression form, and it remained elevated after re-hydration (Figure 5A). A higher, compared with the control, activity of APX was noticed under advanced drought (D3) and after re-hydration in the case of the HDT form (Figure 5C). The activity of GPX was constant in the HDT introgression form during the whole experiment but it increased gradually under stress conditions in the LDT form and achieved the level observed in the HDT form at D2 (Figure 5B). After re-hydration, the LDT form maintained this elevated activity. A higher activity of GR under water deficit was visible at D1 in the LDT introgression form compared with the HDT form (Figure 5D). The activity of SOD did not show any statistically significant changes during drought treatment in both introgression forms, though some slight disturbances with respect to this parameter were observed. Significantly higher SOD activity after re-hydration was observed in the LDT form compared with the other introgression form (Figure 5E).

### 2.8. Superoxide Anion Radical and Hydrogen Peroxide Content

**Figure 6:**
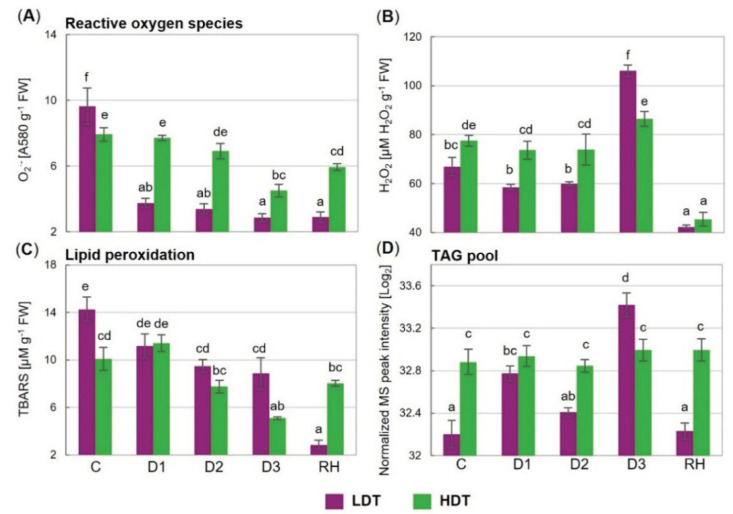
(**A**) Content of superoxide anion radical (O_2_^•−^), (**B**) hydrogen peroxide (H_2_O_2_), (**C**) thiobarbituric-reactive substances (TBARS), and (**D**) triacylglycerol lipid pool (TAG pool) in two *L. multiflorum/F. arundinacea* introgression forms (low- and high-drought tolerant—LDT and HDT, respectively) before stress treatment (C), on the 3rd (D1), 6th (D2), and 10th (D3) day of the water deficit and 10 days after re-hydration (RH). Error bars represent the standard errors. Homogeneous groups are denoted by the same letter according to Fisher’s LSD test (*p* = 0.01). FW—leaf fresh weight.

A considerable decrease in the superoxide anion radical content (more than 2.5-fold), at the beginning of stress treatment (D1), was noticed in the LDT introgression form (Figure 6A). This lower level was maintained during prolonged water deficit and also after re-hydration. The content of O_2_^•−^ was also reduced under drought in the HDT introgression form, but only in the severe stage (D3) (Figure 6A). During the whole period of stress and also after re-watering, the level of O_2_^•−^ was higher in the HDT form, whereas under the control conditions the opposite was observed.

The advanced drought caused an increase in the hydrogen peroxide content, compared to the control, in the LDT introgression form (Figure 6B). In the control and initial drought period, the content of H_2_O_2_ was higher in the HDT form. On the other hand, on the 10th day of stress duration, its accumulation level was relatively higher in the LDT form. After re-watering, the H_2_O_2_ content was reduced drastically in both introgression forms, and no differences between the analyzed plants were observed.

### 2.9. Lipid Peroxidation Level

The introgression forms differed in the thiobarbituric-reactive substance (TBARS) content under the control conditions and on the 10th day of the water deficit, where their accumulation levels were higher in the LDT form (Figure 6C). Under advanced stress conditions, at D2 in the case of the LDT form, and at D3 in the case of the HDT form, a reduction in the TBARS content, compared with the control values, was visible. Moreover, the TBARS content decreased subsequently in the LDT form after re-watering.

### 2.10. Triacylglycerol Lipid Accumulation

In the HDT form, the content of the TAG pool was constant throughout the whole experiment. On the other hand, the LDT introgression form showed a changing pattern in the accumulation profile of this class of lipids (Figure 6D). Under the control conditions, at the D2 and RH time points, the TAG content was significantly higher in the HDT form, while at D3 it was higher in the LDT form. After re-watering, the TAG content returned to the control level in the case of the LDT form but remained at the same level in the HDT introgression form.

## 3. Discussion

### 3.1. Plant Selection Following Prolonged Drought and Subsequent Re-Watering under Field Conditions

In this study, two *L. multiflorum/F. arundinacea* introgression forms with a different ability to survive prolonged water deficit conditions in the field were comprehensively researched to identify the crucial components of cellular metabolism involved in the mechanisms of drought tolerance in forage grasses. Although both introgression forms revealed comparable yield potential in the field, under simulated stress conditions, they differed significantly in their capacity to re-grow after stress cessation. The LDT form did not survive the drought period followed by re-watering, while the HDT form demonstrated a relatively high level of drought tolerance. Our earlier research performed under similar environmental conditions (see Perlikowski et al. (2014) [46]) revealed that two other introgression forms, besides possessing different abilities to re-grow after re-watering, were also characterized by different yield potential during water deficit conditions. Further work proved that this was mainly due to the drought avoidance strategy developed by the HDT form, and that phenomenon was associated with a different capacity of the analyzed introgression forms to develop deep root systems [40]. In the current experiments, the survival strategy of the HDT form seemed to be, at least partially, different. First, we could not exclude here that both introgression forms, the HDT and LDT, were able to develop deep root systems under drought, although a reduction of their yield was also observed, at least at some time points under stress conditions. On the other hand, we also could not exclude that some alterations in root metabolism existed in the case of the LDT form, which had significantly lower survival. However, we assumed here that disturbances in leaf cellular metabolism of the LDT form could result in death after stress termination and/or that adaptations in leaf cellular metabolism of the HDT form could result in its survival advantage. *L. multiflorum*/*F. arundinacea* introgression forms, with the selected components of stress tolerance transferred from *F. arundinacea* to the *L. multiflorum* genomic background, drought tolerance [32,40,46,47], and frost tolerance [48,49,50], have been proved to be valuable tools to dissect these complex traits into the different components present in the different introgression lines and further to analyze these components in detail. Thus, herein we applied another pair of introgression forms, which could serve as useful plant models to identify yet unrecognized cellular components involved in the mechanisms of drought tolerance in forage grasses. To approve our hypothesis, we performed the research on the HDT and the LDT introgression forms selected in the field, applying short-term drought treatment in pots, thus in environmental conditions preventing unlimited root development, similarly to our earlier studies [45,46].

### 3.2. Physiological Response to Short-Term Drought

During the short-drought period applied in the environmental chamber, both introgression forms revealed symptoms of physiological disturbances, including loss of leaf turgor (Figure S2). However, we did not observe leaf rolling in the case of these plants, which was shown to be a characteristic trait of *F. arundinacea* and *F. glaucescens* under the same stress conditions [45]. Although RWC in the HDT and LDT plants decreased at the advanced stage of the drought, which was previously observed in *F. arundinacea* and *F. glaucescens* [45], and in the other *L. multiflorum/F. arundinacea* introgression forms [46], the analyzed forms did not differ in the intensity of water loss. It was proved earlier that the RWC analyzed during short-term drought conditions in pots is not always a good indicator of plant yield potential under prolonged drought conditions in the field. Herein, the HDT and LDT forms revealed the same levels of RWC under advanced short-term drought and similar yield parameters under prolonged drought. However, the study of Perlikowski et al. (2014) [46] showed quite a different relationship. The HDT *L. multiflorum/F. arundinacea* introgression form with higher yield under drought in the field had slightly lower RWC under advanced short-term drought in pots. Despite similarities observed in the current research in the dynamics of RWC between the HDT and LDT introgression forms, stress-induced membrane damage was revealed only in the LDT form. This fact could suggest that not only dehydration of leaf tissue *per se* was responsible for this phenomenon but the other factors associated with alterations in cellular metabolism under drought conditions could have a significant impact on the level of membrane integrity in the LDT form. On the other hand, the relationship between RWC and EL in our earlier research performed on *L. multiflorum/F. arundinacea* introgression forms was different. Despite slight differences noticed in RWC values between the two analyzed plants, both had the same levels of electrolyte leakage and membrane damage at the advanced time point of the drought treatment. However, only the HDT form was able to regenerate its membranes after stress cessation, though each analyzed form increased its RWC to the control levels [46]. Herein the EL parameter followed RWC dynamics, and the LDT introgression form regenerated its cellular membranes after stress cessation. The levels of TBARS in both *L. multiflorum/F. arundinacea* introgression forms at the advanced time point (D3) of the drought period were lower compared with the control conditions but simultaneously significantly higher in the LDT form compared with the HDT form. This phenomenon could indicate that the membrane injuries observed in the LDT form at this time point of drought could be only partially associated with lipid peroxidation. An unchanged level of lipid peroxidation and a higher level of EL under drought conditions were previously reported in a *Triticum aestivum* cultivar with high drought tolerance [51]. Peroxidation of leaf membrane lipids increased under prolonged drought in stress-resistant and stress-susceptible genotypes of *L. multiflorum* [52], whereas it remained unchanged in *T. durum* [53] and *Poa pratensis* [54]. Interestingly, after stress cessation in the current study, levels of TBARS dropped drastically but only in the LDT introgression form. To explain the observed differences in TBARS accumulation between the analyzed plants during the whole experiment, further research would be required; however, it is highly probable that the phenomenon is associated with the differences in levels of ROS accumulation between these two introgression forms revealed here. On the other hand, lower lipid peroxidation under advanced drought (D3) in the HDT form could indicate a higher antioxidative capacity, simultaneously reflecting higher drought tolerance of this form, as suggested earlier for *T. aestivum* by Shao et al. (2005) [55]. ABA increased earlier in the HDT form and this form also had higher accumulation of ABA during the whole drought period compared with the LDT form, which in turn demonstrated an elevated ABA content only at the D3 time point. This phenomenon could indicate faster and also more efficient stress signaling in the HDT form. Our previous research on alterations in the leaf lipidome under drought conditions in the other *L. multiflorum/F. arundinacea* introgression forms also suggested that faster stress signaling could be an important attribute of the HDT form [32]. On the other hand, genotypes of *F. arundinacea* distinct in their levels of drought tolerance showed no differences with respect to ABA dynamics and contents under similar experimental conditions [45]. Interestingly, only the LDT genotype of *F. glaucescens* revealed a significant increase in ABA accumulation at the advanced time point of the drought period [45]. Thus, we demonstrated here that the introgression forms investigated in the current research were characterized by unique physiological traits compared with the other plant materials used in our earlier studies and could be valuable models to deepen our understanding of drought tolerance and to indicate novel components of this tolerance in forage grasses.

### 3.3. Gas Exchange, Stomata, and Photosynthetic Performance

The analysis of gas exchange parameters at the advanced time point of drought (D3) did not reveal any significant differences between the analyzed introgression forms except for the internal CO_2_ concentration. The value of this parameter under drought was significantly lower in the HDT form compared with the LDT form. As stomatal conductance was reduced significantly under advanced drought to the same levels in both introgression forms, these relations regarding *Ci* could indicate, at least partially, a higher level of carboxylation in the case of the HDT introgression form. However, this aspect of a plant’s reaction to stress conditions was not fully recognized here. On the other hand, lower non-stomatal photosynthetic limitations in the HDT form were also clearly visible after stress cessation since a significant increase in CO_2_ assimilation was not associated with an increase in g_s_ in that introgression form. A clear relationship between stomatal conductance and the CO_2_ assimilation level under control and drought conditions was observed in our study on the HDT and LDT genotypes of *F. arundinacea* and *F. glaucescens* [45]. A decrease in A and g_s_ during drought was observed also in *Hordeum vulgare* exposed to drought, salinity, and both these stress factors simultaneously [56], which indicated dominance of stomatal factors in photosynthetic regulations in these species. On the other hand, in the work of Perlikowski et al. (2014) [46] performed on *L. multiflorum/F. arundinacea* introgression forms, these relations were noticed only under the control conditions in both forms distinct in their levels of drought tolerance. However, under drought, it was the LDT form that had higher CO_2_ assimilation, having simultaneously the same values of g_s_, as the HDT form. Moreover, a higher rate of photosynthesis observed in the LDT form under control conditions was associated with higher accumulation of metabolites in this form, including TAG lipids [46]. Thus, it is highly probable that the observed here higher accumulation of TAG under control conditions in the HDT form could be associated with a higher photosynthetic capacity of this introgression form, manifested by higher assimilation of CO_2_. It was suggested earlier that carbon metabolism could trigger TAG accumulation in plants. This phenomenon was reported previously for forage grasses [32,57,58].

In both introgression forms, stomatal density did not change considerably during drought, while stomatal size was reduced in the LDT form. *Arabidopsis* plants grown under water restriction did not show alterations in SD; however, reductions in SS were observed [59]. Interestingly, clear differences in SD, SS, and SCs were noticed between the analyzed introgression forms in the current study. The LDT form had significantly higher stomatal density than the HDT form. Recent studies demonstrated that a reduction in SD by overexpression of EPF1 (epidermal patterning factor) in *H. vulgare* [60] and *Oryza sativa* [61] improved their drought tolerance. However, under water-restricted conditions in two *H. vulgare* transgenic lines (which displayed ∼47% and 0.6% SD of controls), there were no differences in WUEi in comparison to the control conditions [60]. Similarly, in the LDT and HDT introgression forms, despite the significant distinction in SD, no differences in WUEi under drought were observed. This phenomenon could be explained, at least partially, by clear differences with respect to subsidiary cell size and stomatal size noticed between the HDT and LDT form. The HDT form had lower stomatal density but simultaneously larger subsidiary cells and stomatal size. Thus, consequently, the level of particular parameters of gas exchange, including g_s_, A, and E under stress conditions was similar in both analyzed introgression forms. In grasses, highly modified lateral subsidiary cells play an important physiological role associated with stomatal functions [23]. Mutant plants lacking subsidiary cells failed to open stomata with guard cells as widely as control plants and also showed slower stomatal responses to changes in light intensity. These facts suggested that subsidiary cells are crucial for efficient stomatal functioning in grasses [62]. During evolutionary processes, grasses developed stomata composed of a pair of dumbbell-shaped guard cells flanked by paracytic subsidiary cells. This anatomical achievement allows a quick and more dynamic response to environmental stimuli compared with dicots. It has been suggested that the shape and volume of subsidiary cells can significantly affect stomata opening in both physical (turgor-driven mechanics) and chemical ways (ion transport), influencing their potential to open or close under stress conditions [23]. A precise relationship between subsidiary and guard cells is still unclear; however, this relation can be considered an important factor influencing a plant’s capacity to withstand adverse environmental conditions. As mentioned earlier, the HDT introgression form, which had significantly lower stomatal density, developed larger subsidiary cells. There is some evidence to support a negative relationship between stomatal density and stomatal size across a range of plants [25,59], which was also demonstrated for our introgression forms. Precise understanding of this phenomenon in grasses needs further detailed functional characterization.

The observed reduction of maximum quantum efficiency of PSII in the HDT and LDT forms under drought was transient and this parameter returned to the control levels after re-watering in both introgression forms. However, as indicated earlier, Fv/Fm and the other parameters of chlorophyll fluorescence under drought conditions could be more often associated with the water content in plant tissue but not always with photosynthetic activity [63]. In our earlier study on *L. multiflorum/F. arundinacea* introgression forms, we noticed that plants with different photosynthetic activities under drought did not differ with respect to parameters of chlorophyll fluorescence [46]. Therefore, these parameters cannot be regarded as good indicators of the photosynthetic apparatus response to water deficit. On the other hand, the adaptation of the Calvin cycle to drought conditions and to reduced CO_2_ availability was more visible in the HDT form. First, despite reduced CO_2_ assimilation after water deficit initiation, the accumulation of pFBA significantly increased in the LDT form but remained constant in the HDT form, compared with the control conditions. Additionally, although no differences with respect to CO_2_ assimilation under drought were observed between the analyzed introgression forms, both the accumulation and activity of pFBA were significantly higher in the LDT form. Finally, although aldolase activity dropped gradually under drought conditions in both analyzed introgression forms, the reduction of this activity was faster in the case of the HDT form. This parameter reached its lowest value at the D2 time point in the HDT and at D3 in the LDT form. We cannot exclude that this phenomenon could be partially associated with the observed differences between the analyzed plants with respect to stress signaling and ABA accumulation, which was also faster and higher in the case of the HDT form. The activity of pFBA was proved earlier to be a valuable marker of the Calvin cycle efficiency under drought conditions in *Lolium-Festuca* grasses [45,47]. Interestingly, higher accumulation and activity of pFBA under water deficit, applied under similar experimental conditions, was observed earlier for the LDT form in the other pair of *L. multiflorum/F. arundinacea* introgression forms [47]. However, in that case, this phenomenon was tightly associated with a higher assimilation of CO_2_ under drought in the LDT form, compared with the HDT form, and consequently, a higher efficiency of the Calvin cycle was proved to be a crucial metabolic component reducing non-stomatal limitations of photosynthesis [46,47]. Herein, stable accumulation of plastid fructose-1,6-bisphosphate aldolase and a faster decrease of its activity, observed in the HDT introgression form under drought, seemed to be a functional adjustment to limited availability of CO_2_ due to stomatal closure. We cannot exclude that this adjustment could also be important under prolonged drought conditions in the field and that it could be a crucial component of the survival strategy of forage grasses. A reduced abundance of pFBA under water deficit was demonstrated in drought-tolerant, compared to drought-sensitive, genotypes of *H. vulgare* DH lines [28], drought-tolerant and drought-sensitive cultivars of *T. aestivum* [64], and in *Glycine max* [65]. Plastidial PGK2 was shown earlier to play an important role in the expression of tolerance to abiotic stresses. An over-expression of AtPGK2 in *Arabidopsis* [66] and OsPgk2a-P [67] in transgenic *Nicotiana tabacum* improved their tolerance to salinity stress. In the current research, the activity of pPKG did not reveal any differences between the analyzed introgression forms, showing constant levels in the control and under drought conditions. After re-watering, a clear recovery of the Calvin cycle activity was noticed in both introgression forms, manifested by a significant increase in pFBA and pPKG activities. On the other hand, although higher efficiency of the Calvin cycle after re-hydration was accompanied by a higher level of CO_2_ assimilation in both forms, only in the case of the LDT form was it also associated with significant stomatal opening. Thus, photosynthetic recovery after stress cessation was enhanced by both stomatal and non-stomatal factors in the LDT form but only by non-stomatal factors in the HDT form. Furthermore, the activity of pFBA after re-watering achieved the level observed under the control conditions in the LDT introgression form but not in the HDT form. All these data indicated that photosynthetic adjustment to water deficit is an important component of plant metabolism under stress and recovery periods in forage grasses. Moreover, we revealed that the *L. multiflorum/F. arundinacea* introgression forms distinct in their levels of drought tolerance under different experimental conditions also differed in their capacity to adapt their photosynthetic apparatus to stress treatments. Decreased photosynthesis under severe drought was caused by stomatal limitations in *O. sativa* [68], while in *Medicago sativa,* it was an effect of non-stomatal limitations [69].

### 3.4. Reactive Oxygen Species and Antioxidant Capacity

Disrupted photosynthesis caused by water deficit usually results in ROS overproduction which induces oxidative stress [70]. In our study, accumulation of hydrogen peroxide increased significantly only in the LDT introgression form under advanced drought (D3), compared to the control conditions. The increase in H_2_O_2_ at D3, in the case of the HDT form, was slight, compared to the earlier time points of water deficit but statistically not significant compared with the control time point. However, no clear and significant differences in CAT, APX, GPX, and GR activities were found between the analyzed introgression forms at a majority of the experimental time points, and especially at the D3 time point of drought application. Moreover, the accumulation of these enzymes was equal at all time-points of the drought period in both analyzed introgression forms. From this point of view, that phenomenon could mean that the capacity of the enzymatic antioxidant system *per se* was not involved in the process to maintain the observed H_2_O_2_ balance under drought and re-watering conditions in the HDT and LDT forms; however, we cannot exclude that a higher activity of GPX in the control and on the 1st day of drought in the HDT form could also be associated, at least partially, with this process. On the other hand, a progressive increase in CAT and APX activities was revealed during the drought period in the HDT form, and these activities were significantly higher at the D3 time point in this introgression form, compared with the control conditions. Such dynamics of CAT and APX activities was not observed in the LDT form. This phenomenon could be responsible for the maintenance of a stable hydrogen peroxide content under the control and stress conditions in the HDT introgression form. Higher CAT activity under drought was reported for different species, including *Zea mays* [71], *T. aestivum* [72], *L. multiflorum* [52], and *F. glaucescens* [45]. By contrast, no significant changes under drought in leaf CAT activity were observed in *P. pratensis* [54] and *F. arundinacea* [45]. By maintaining a stable level of H_2_O_2_ and the level of free transient metals such as Fe^2+^ under control, plant cells are able to prevent the formation of the highly toxic hydroxyl radical (HO^•^) via the Fenton reaction and consequently to reduce DNA damage, protein oxidation, and lipid peroxidation [73]. Similar to our observations, the accumulation of hydrogen peroxide under water deficit in *P. pratensis* did not contribute directly to lipid peroxidation [54]. In plants exposed to abiotic stresses, an elevated level of the superoxide anion radical was reported [28,56]. During water deficit and re-watering periods, the content of O_2_^•−^ was significantly lower, compared with the control, in both introgression forms. This phenomenon was accompanied by a relatively high level of lipid peroxidation in the control conditions compared to the D2–D3 time points in the LDT, and to D3 in the HDT form. However, we are unable to explain the physiological and molecular background of that phenomenon. The analyzed plants grew for two weeks in the environmental chamber under control conditions before the water deficit was initiated in the same chamber, and the last day of that 2-week period was applied as the control in the current study. On the other hand, a higher content of O_2_^•−^ under drought and re-hydration was revealed for the HDT introgression form, compared to the LDT form. Under re-watering conditions, higher SOD activity was in fact noticed in the LDT form, but on the other hand, no significant differences in SOD activity under water deficit between the analyzed forms were observed in our study. Depending on the metal bound in the catalytic active site, three SOD isoforms are distinguished in plants: manganese SOD (Mn-SOD), copper/zinc SOD (Cu/Zn-SOD), and iron SOD (Fe-SOD). Isoforms are located in different cellular compartments. Fe-SOD is mainly located in the chloroplast, Mn-SOD in mitochondria and peroxisomes, while the most plentiful SOD form in plant cells, Cu/Zn-SOD, is detected in numerous cell compartments: chloroplasts, mitochondria, cytosol, peroxisomes, and the apoplast [74,75]. Cu/Zn-SOD and Fe-SOD isoforms revealed a higher accumulation level under advanced drought in the HDT introgression from. Unchanged by drought stress, SOD activity was reported for *P. pratensis* [54], whereas it decreased in *O. sativa* [76] and *M. sativa* [77]. A higher content of O_2_^•−^ in the HDT form, and its limited dismutation into O_2_ and H_2_O_2_, could have helped to prevent an increase in H_2_O_2_ in this introgression form. A specific balance of hydrogen peroxide under stress conditions is also important with respect to the regulation of gene expression, which could be crucial to develop stress tolerance [78]. It should also be noted that drought signaling is interlinked with ROS and nitric oxide [79], so the observed stress-reduced superoxide formation may be associated with peroxynitrite formation that might act as a powerful modulator of cellular redox balance towards tolerance. Accumulation of hydrogen peroxide observed in the LTD introgression form under advanced drought could be associated with a synthesis of TAG lipids that are suggested to play an important role in the neutralization of oxidized FA [80]. It was hypothesized that the accumulation of TAG may function as a mechanism for dissipation of excess radiation energy in leaves [27], and can be associated with a stabilization of membranes during dehydration [81,82]. Moreover, accumulation of TAG lipids could help to accommodate a shrinking organelle during osmotic stress by removing excess lipids from the membranes [81] and protecting cells against lipid peroxidation and ROS-induced oxidative damage by converting free FA and damaged lipids into TAG [34,83]. A stable level of TAG in the HDT form, compared to the LDT form, might be due to a higher capacity of the antioxidant system in the form with higher drought tolerance.

## 4. Materials and Methods

### 4.1. Plant Materials

The population of the *L. multiflorum/F. arundinacea* introgression forms was obtained through backcrossing the *L. multiflorum* (4x) × *F. arundinacea* (6x) hybrid to *L. multiflorum* (4x) [46]. Two introgression forms significantly differing in the level of drought tolerance, high-drought-tolerant (HDT) and low-drought-tolerant (LDT), were used in the current study. These plants were selected following long-term drought in the field under ‘rain-out’ shelters at Danko Plant Breeding Ltd., in Szelejewo, Poland (51°54′00″ N; 17°12′00″ E). The procedure of field experiments was described by Perlikowski et al. (2014) [46].

### 4.2. Plant Selection with Respect to Drought Tolerance under Field Conditions

A long-term drought experiment was performed under ‘rain-out’ shelters [46]. After establishment, a population of 140 individuals was exposed to a 13-week drought followed by 3-week re-watering (June–September 2014). Plants were cut, and their regrowth and further bonitation abilities were analyzed (Figure S1A). Re-growth and bonitation of tillers were evaluated on a scale, 1–9, after plant installation, at three different time points of the water deficit (on the 13th, 29th, and 53rd and the 18th, 36th, and 65th day for re-growth and bonitation, respectively) as well as after re-watering. For each individual and parameter, a mean score (arithmetic mean of three scores derived from three clones from three distinct shelters) was calculated. The ambient temperature and rainfall were monitored. 

### 4.3. Short-Term Drought Conditions in the Environmental Chamber

The HDT and LDT introgression forms were exposed to short-term drought performed in the environmental chamber as described by Kosmala et al. (2012) [44]. The experimental period covered two weeks for plant installation, followed by 10-day drought and further 10-day re-hydration in the same chamber under the following conditions: 22 °C, 400 μmol m^−2^ s^−1^ (photosynthetic photon flux density), 16/8 h for the day/night cycle and 50–60% air humidity. Soil water capacity (SWC) during drought stress was monitored for each plant through daily weighing. Leaf material was harvested one day before the stress treatment, following a 2-week period of plant installation (control, C) and at three different time points of the water deficit on the 3rd (D1), 6th (D2), and 10th (D3) day, as well as 10 days after subsequent re-watering (RH).

### 4.4. Physiological Parameters

Physiological parameters including the relative water content (RWC), electrolyte leakage (EL), chlorophyll ‘a’ fluorescence, and gas exchange: (CO_2_ assimilation (A), transpiration (E), stomatal conductance (g_s_), internal CO_2_ concentration (*Ci*)) were measured as described previously in detail by Kosmala et al. (2012) [44], Perlikowski et al. (2014) [46], and Lechowicz et al. (2020) [45]. Intrinsic water use efficiency (WUEi) was calculated as the ratio of A to g_s_ [84]. EL was measured using a Hanna Instruments EC215 Multi-range Conductivity Meter (Rhode Island, USA), whereas chlorophyll ‘a’ fluorescence was measured using a HandyPEA fluorimeter (Hansatech Instruments Ltd., King’s Lynn, UK) at midday. A CIRAS-2 Portable Photosynthesis System (Hitchin, UK) was used for gas exchange measurements. RWC, EL as well as chlorophyll ‘a’ fluorescence were measured at five time points (C, D1, D2, D3, and RH) (ten individual measurements per time point per introgression form), whereas gas exchange was measured at three time points (C, D3, and RH) (five individual measurements per time point per introgression form). The second fully expanded leaves were used for all physiological analyses; for gas exchange, the middle part of the leaves was used. The ABA content was measured with a Microplate Reader Model 680 (Bio-Rad, Hercules, CA, USA) using a Plant Hormone Abscisic Acid ELISA Kit (CSB-E09159Pl, CUSABIO, Wuhan, China), according to the manufacturer’s instructions. Three biological replicates, represented by pooled leaves collected at five time points (C, D1, D2, D3, and RH), were used.

### 4.5. Anatomy and Density of Stomata

The middle part of the second fully expanded leaves (1 cm fragments, from the same region) was fixed in 3 volumes of 98.8% ethanol and 1 volume of 100% acetic acid for 24 h, then cleared and mounted in 100% lactic acid until the tissue became transparent. Cell images of the abaxial epidermis were acquired under an M2 motorised Zeiss microscope (Carl Zeiss Microscopy GmbH, Göttingen, Germany) under Nomarski’s DIC contrast. Approximately 30–50 stomata were drowned using an LCD tablet (Wacom drawing pad), and the ImageJ image analysis software [85]. Stomatal density (SD), expressed as the number of stomata per 1 mm^2^, was calculated by dividing the number of stomata by the area of the analyzed epidermal tissue. Additionally, the stomatal size (SS) and size of subsidiary cells (SCs) were checked. The analysis was performed at three time points (C, D3, and RH) in three replicates.

### 4.6. RT-qPCR of the Calvin Cycle and Antioxidant Enzymes

RT-qPCR analyses were carried out for two enzymes of the Calvin cycle: plastid fructose-1,6-bisphosphate aldolase (pFBA) and plastid phosphoglycerate kinase (pPGK) as well as for seven antioxidant enzymes: chloroplastic glutathione reductase (GR), chloroplastic glutathione peroxidase (GPX), chloroplastic Fe-dependent superoxide dismutase (Fe-SOD), chloroplastic Cu/Zn superoxide dismutase (Cu/Zn-SOD), mitochondrial manganese superoxide dismutase (Mn-SOD), chloroplastic L-ascorbate peroxidase (APX), and catalase (CAT). Actin and ubiquitin were used as reference genes. RNA extraction, cDNA synthesis, and RT-qPCR assays were precisely described by Lechowicz et al. (2020) [45]. Sequences of primers and TaqMan probes of the analyzed genes, designed based on the gene cDNA sequences through Beacon Designer software, are presented in Table 1. Normalized gene expression was calculated using the ΔΔCq method. Three biological replicates, represented by pooled leaves collected at five time points (C, D1, D2, D3, and RH), were used.

### 4.7. Western Blot of the Calvin Cycle and Antioxidant Enzymes

Protein accumulation profiles of the Calvin cycle enzymes, pFBA, pPGK, and antioxidant enzymes, chloroplastic and cytosolic glutathione reductase (GR), chloroplastic glutathione peroxidase (GPX), chloroplastic iron superoxide dismutase (Fe-SOD), chloroplastic copper/zinc superoxide dismutase (Cu/Zn-SOD), mitochondrial manganese superoxide dismutase Mn-SOD, chloroplastic/thylakoid L-ascorbate peroxidase (APX), and catalase (CAT), were analyzed. Total proteins were extracted using the Hurkman and Tanaka protocol [86] with slight modifications described earlier by Lechowicz et al. (2020) [45], whereas Western blot assay was performed as described by Pawłowicz et al. (2012) [87]. For immunodetection, specific antibodies were applied (Agrisera, Vännäs, Sweden). Antibodies against the Calvin cycle enzymes: anti-pFBA was produced as described by Perlikowski et al. (2016) [47], and anti-pPGK, as described by Lechowicz et al. (2020) [45]. For antioxidant enzymes, commercial polyclonal antibodies (GR – AS06 181; GPX – AS0 055; Fe-SOD – AS0 125; Cu/Zn-SOD – AS06 170; Mn-SOD – AS09 524; APX – AS08 368; CAT – AS09 501, Agrisera, Vännäs, Sweden), were applied. The APX antibody was diluted at 1:2000, while the remaining antibodies were diluted at 1:4000. Antigen–antibody complexes were detected using a secondary anti-rabbit IgG–horse radish peroxidase conjugate (Sigma, St. Louis, MO, USA, currently member of Merck Group, Darmstadt, Germany) diluted 1:20,000 (1 h of incubation). Chemiluminescent substrates Westar Supernova (Cyanagen, Bologna, Italy) and ChemiDoc^TM^ Touch Igmagin System (Bio-Rad, Hercules, CA, USA) were used to visualize the results. The intensities of visualized bands were estimated using ImageJ software. All measurements were carried out at five time points (C, D1, D2, D3, and RH) in three biological replicates represented by pooled leaves.

### 4.8. Activity of the Calvin Cycle Enzymes

Chloroplast proteins were extracted using a Chloroplast Isolation Kit (Sigma, St. Louis, MO, USA, currently member of Merck Group, Darmstadt, Germany) as described by Kosmala et al. (2012) [44] and Perlikowski et al. (2016) [47] with slight modifications precisely described by Lechowicz et al. (2020) [45]. The chloroplast proteins were dissolved in 0.1 M phosphate buffer (0.1 M Na_2_HPO_4_) with 3% Triton X100, and centrifuged at 21,500× *g* for 10 min in 4 °C. The collected supernatant was used to determine enzyme activity. For normalization, the amount of isolated chloroplasts in the samples was checked spectrophotometrically at 652 nm using 10 µL of the chloroplast suspension and 1 mL of 80% acetone solution. The activity of pFBA was measured according to the modified method of Sibley-Lehninger [88,89] shown in detail by Lechowicz et al. (2020) [45]. In the case of pFBA activity, the amount of produced trioses was estimated with the use of a standard curve and expressed as µg of D-glyceraldehyde produced by 1 g of plant sample during 1 h. The activity of pPGK was measured using the Phosphoglycerate Kinase Activity Assay Kit (ab252890, Abcam, Cambridge, UK), according to the protocol with some modifications. pPGK activity was expressed as µmol of 1,3-bisphosphoglycerate generated by the enzyme from 1 g of plant sample during 1 h at pH 7.2 and 37 °C. The activities of pFBA and pPGK were analyzed with an Ultrospec 1100pro (Amersham Biosciences, Chalfont St. Giles, UK) and a Synergy HTX Multi-Mode Reader (BioTek Instruments, Winooski, Vermont, USA), respectively, at five time points (C, D1, D2, D3, and RH) in three biological replicates represented by pooled leaves.

### 4.9. Activity of Antioxidant Enzymes

The total activity of GPX, GR, SOD, and APX was assayed using the following kits: Glutathione Peroxidase Assay Kit (ab102530, Abcam, Cambridge, UK), Glutathione Reductase Assay Kit (ab83461, Abcam, Cambridge, UK), Superoxide Dismutase Activity Assay Kit (ab65354, Abcam, Cambridge, UK), and Ascorbate Peroxidase Microplate Assay Kit (CAK1052, Cohesion Biosciences, UK), respectively, according to the manufacturers’ protocols. Extracts were prepared from pooled leaves of each biological replicate. One unit of GPX activity was defined as the amount of enzyme that causes the oxidation of 1 µmol of NADPH to NADP^+^ at 25 °C per minute. One unit of GR activity was defined as the amount of enzyme that generates 1 μmol of nitrobenzoic acid (TNB) per minute at 25 °C. One unit of SOD activity represented the amount of enzyme that inhibits 50% of xanthine oxidation at 37 °C. One unit of APX activity was the amount of enzyme that oxidizes 1 μmol ascorbic acid per minute. CAT activity was estimated following the method of Dhindsa et al. (1981) [90], described in detail by Lechowicz et al. (2020) [45]. One unit of CAT activity was defined as the amount of enzyme catalyzing the decomposition of 1 µmol H_2_O_2_ per minute calculated from the extinction coefficient 45.2 mM^−1^ cm^−1^. To normalize the values of activity obtained for each enzyme, the soluble protein content in all the samples was determined according to Bradford (1976) [91]. The activity measurements were performed with a Synergy HTX Multi-Mode Reader (BioTek Instruments, Winooski, Vermont, USA) at five time points (C, D1, D2, D3, and RH) in three biological replicates represented by pooled leaves.

### 4.10. TBARS Content

The content of thiobarbituric-reactive substances (TBARS) in the samples was measured spectrophotometrically at 532 nm and at 600 nm, according to the method of Heath and Packer (1968) [92] with slight modifications [45]. The amount of TBARS was calculated through the following formula: TBARS (µM) = (A_532_–A_600_)/155, where 155 is an extinction factor, expressed per 1 g of fresh sample weight (FW). The analysis was performed at five time points (C, D1, D2, D3, and RH) in three replicates.

### 4.11. Superoxide Anion Radical and Hydrogen Peroxide Content

The superoxide anion radical (O_2_^•−^) was assayed spectrophotometrically according to Doke (1983) [93], and Arasimowicz et al. (2009) [94] with slight modifications [45]. Fresh leaf discs (0.6 cm in diameter) were incubated with 3 mL of 0.05 M potassium-phosphate buffer (pH 7.8), containing 0.1 mM EDTA; 10 mM NaN_3,_ and 0.05% nitrotetrazolium blue chloride (NBT) for 1 h in the dark. Then, the samples were incubated at 85 °C for 15 min. After cooling on ice, the absorbance was measured at 580 nm. The level of O_2_^•−^ was expressed as the absorbance of the sample measured at 580 nm per 1 g of FW. Hydrogen peroxide (H_2_O_2_) was measured spectrophotometrically using the titanium (Ti4^+^) method [95,96] described in detail by Lechowicz et al. (2020) [45]. The absorbance was measured at 508 nm after a 10-min incubation. The level of H_2_O_2_ was determined based on a standard curve and expressed as µM of H_2_O_2_ per 1 g of FW. Both analyses were performed at five time points (C, D1, D2, D3, and RH) in four (O_2_^•−^) and three (H_2_O_2_) replicates.

### 4.12. TAG Accumulation

In total, 25 mg of lyophilized leaf tissue was used for lipid extraction in 1 mL of a precooled (15 °C) mixture of methyl-tert-butyl ether (3:1:1, v/v/v; MTBE) and separated by a reversed-phase C8 column on an Acquity UPLC system (Waters, Milford, Massachusetts, USA). Lipids were further identified using an Exactive high resolution mass spectrometer (Thermo Fisher, Waltham, MA, USA) and annotated according to the retention time and the mass-to-charge ratio (m/z) by querying against the in-house database created from 13C isotope-labeled lipids extracted from *Arabidopsis thaliana* [97,98], as described previously by Perlikowski et al. (2016) [32]. The analysis was performed at five time points (C, D1, D2, D3, RH) in three biological replicates represented by pooled leaves.

### 4.13. Statistical Analysis

Statistical analyses were performed by using STATISTICA 13.1 software (StatSoft, Tulsa OK, USA). Two-way analysis of variance (ANOVA), with introgression form and time point as classification factors, was conducted. Differences between the plants during the experiment were evaluated using Fisher’s least significant difference (LSD) test at *p* = 0.01. Homogeneous groups were denoted by the same letter in the graphs.

## 5. Conclusions

The HDT introgression form with the ability to recover after prolonged drought period in the field was also characterized by earlier and higher accumulation of ABA, more stable cellular membranes, a higher adaptation of its photosynthetic apparatus and enzymatic antioxidant system to water deficit, and consequently more balanced ROS metabolism under short-term drought in pots. On the other hand, though different sizes and densities of stomata and sizes of subsidiary cells were observed between the analyzed introgression forms, both the HDT and LDT plants revealed similar levels of stomatal conductance, CO_2_ assimilation and consequently, WUEi under short-term drought. However, the adjustment of the Calvin cycle to water deficit conditions and to reduced CO_2_ availability, with respect to the accumulation and activity of plastid fructose-1,6-bisphosphate aldolase, was more visible in the HDT form. Thus, we assume that this adjustment could also be important under prolonged drought conditions in the field and that it could be a crucial component of the survival strategy of forage grasses. The increasing accumulation of TAG in the LDT introgression form, particularly at the advanced stage of drought, could be associated, at least partially, with the initiation of processes triggering a reduction of oxidative stress. By contrast, the HDT introgression form, characterized by a higher activity of antioxidant enzymes, associated with scavenging of hydrogen peroxide and consequently by its lower level under advanced drought, maintained a stable level of TAG. However, this phenomenon has not been well-recognized in plants and requires further research.

## Figures and Tables

**Table 1:** Primers, probe sequences, and amplicon lengths of reference and target genes used for RT-qPCR analyses.

Gene	Primer Forward/Reverse	TaqMan Probe	Amplicon Length (bp)
Actin	GTCGAGGGCAACATATGCAA CCAGTGCTGAGCGGGAAT	TTCTCCTTGATGTCACGGAC	65
Ubiquitin	GCAAGAAGAAGACGTACA GACCTTGTAGAACTGGAG	CTTCACCTTCTTGTCCTTGTGCTT	86
pFBA	GAGACGTTCTACTACATG GAGGAGCTTGAGAGTGTA	TGTTCCTGTCCTTGCACTCGG	140
pPGK	CCTTGGTTGAGGAAGATAA CAGCAATGACAACATCAG	CTGGCAACAACTCTCCTGGC	102
GR	GGGGAGTACGACTACGACCT TCGTAAGTCCACCCAAAGCC	GGCGGCGTCAGGGCCTCGCGCTT	254
GPX	TCACTCGGCGGCCTGGAGAA TTCACAGTGCGGGCTTACGA	CTACGCCACCGCCGCCACGGAGAA	212
APX	CTCGTATCGCAGGAGCTCG TTGGGCCACTCGCTAATGTT	CGGCTGCGGCTGGAGATGCGACGGC	188
Fe-SOD	TCTATCTCGGCGGTTCTCCA CCGTTGTTGTAGGCCTCCTT	GCTCGACACCAGCCCCTTCTACGGCCA	219
Cu/Zn-SOD	CCAGAGCATCCTCTTCGCC ATTGATGGAGGTGGAAGCCG	TCGCTCCGCCTCGTCTCCGCCCCC	287
Mn-SOD	TTGACGCCGCTGTCTCTAAGG TTTATCCAACGCCAGCCACA	GCTTCCGCCGTCGTCCAACTCCAGGGC	266
CAT	GTTCACCTTCCTCTTCGACG AAGTCGAACCTGTCCTCGTG	ACTACCGCCACATGGATGGCTCCG	297

## Supplementary Materials

Supplementary materials can be found at https://www.mdpi.com/1422-0067/21/16/5639/s1.

## Abbreviations

A	CO_2_ assimilation
ABA	abscisic acid
APX	L-ascorbate peroxidase
CAT	catalase
*Ci*	internal CO_2_ concentration
Cu/Zn-SOD	copper/zinc superoxide dismutase
E	transpiration
EL	electrolyte leakage
FA	fatty acids
Fe-SOD	iron superoxide dismutase
Fv/Fm	maximum quantum efficiency of PSII photochemistry
FW	fresh weight
GC	guard cells
g_s_	stomatal conductance
GPX	glutathione peroxidase
GR	glutathione reductase
HDT	high drought tolerance
LDT	low drought tolerance
Mn-SOD	manganese superoxide dismutase
SC	subsidiary cells
SD	stomatal density
SOD	superoxide dismutase
SS	stomatal size
SWC	soil water capacity
pFBA	plastid fructose-1,6-bisphosphate aldolase
pPGK	plastid phosphoglycerate kinase
PSII	photosystem II
ROS	reactive oxygen species
RWC	relative water content
TAG TBARS	triacylglycerol thiobarbituric-reactive substances
WUEi	intrinsic water use efficiency

## References

[1] Spinoni J., Vogt J.V., Naumann G., Barbosa P., Dosio A. (2018). Will Drought Events Become More Frequent and Severe in Europe?. Int. J. Climatol..

[2] Xu Z., Zhou G. (2008). Responses of Leaf Stomatal Density to Water Status and its Relationship with Photosynthesis in a Grass. J. Exp. Bot..

[3] Flexas J., Niinemets Ü., Gallé A., Barbour M.M., Centritto M., Diaz-Espejo A., Douthe C., Galmés J., Ribas-Carbo M., Rodriguez P.L. (2013). Diffusional Conductances to Co_2_ as a Target for Increasing Photosynthesis and Photosynthetic Water-Use Efficiency. Photosynth. Res..

[4] Wang Z., Li G., Sun H., Ma L., Guo Y., Zhao Z., Gao H., Mei L. (2018). Effects of Drought Stress on Photosynthesis and Photosynthetic Electron Transport Chain in Young Apple Tree Leaves. Biol. Open.

[5] Chaves M.M., Flexas J., Pinheiro C. (2009). Photosynthesis under Drought and Salt Stress: Regulation Mechanisms from Whole Plant to Cell. Ann. Bot..

[6] Soares-Cordeiro A.S., Driscoll S.P., Pellny T.K., Olmos E., Arrabaça M.C., Foyer C.H. (2009). Variations in the Dorso-Ventral Organization of Leaf Structure and Kranz Anatomy Coordinate the Control of Photosynthesis and Associated Signalling at the Whole Leaf Level in Monocotyledonous Species. Plant. Cell Environ..

[7] Pena-Rojas K., Aranda X., Fleck I. (2004). Stomatal Limitation to CO_2_ Assimilation and Down-Regulation of Photosynthesis in Quercus Ilex Resprouts in Response to Slowly Imposed Drought. Tree Physiol..

[8] Lawlor D.W. (2002). Limitation to Photosynthesis in Water-Stressed Leaves: Stomata vs. Metabolism and the Role of ATP. Ann. Bot..

[9] Lawlor D.W., Cornic G. (2002). Photosynthetic Carbon Assimilation and Associated Metabolism in Relation to Water Deficits in Higher Plants. Plant Cell Environ..

[10] Morison J.I.L., Lawson T. (2007). Does Lateral Gas Diffusion in Leaves Matter?. Plant Cell Environ..

[11] Lake J.A., Woodward F.I. (2008). Response of Stomatal Numbers to CO_2_ and Humidity: Control by Transpiration Rate and Abscisic Acid. New Phytol..

[12] Sekiya N., Yano K. (2008). Stomatal Density of Cowpea Correlates with Carbon Isotope Discrimination in Different Phosphorus, Water and Co_2_ Environments. New Phytol..

[13] Fraser L.H., Greenall A., Carlyle C., Turkington R., Friedman C.R. (2009). Adaptive Phenotypic Plasticity of *Pseudoroegneria Spicata*: Response of Stomatal Density, Leaf Area and Biomass to Changes in Water Supply and Increased Temperature. Ann. Bot..

[14] Figueroa J.A., Cabrera H.M., Queirolo C., Hinojosa L.F. (2010). Variability of Water Relations and Photosynthesis in *Eucryphia Cordifolia* cav. (Cunoniaceae) over the Range of its Latitudinal and Altitudinal Distribution in Chile. Tree Physiol..

[15] Yan F., Sun Y., Song F., Liu F. (2012). Differential Responses of Stomatal Morphology to Partial Root-Zone Drying and Deficit Irrigation in Potato Leaves Under Varied Nitrogen Rates. Sci. Hortic. (Amsterdam).

[16] Rudall P.J., Chen E.D., Cullen E. (2017). Evolution and Development of Monocot Stomata. Am. J. Bot..

[17] Hepworth C., Caine R.S., Harrison E.L., Sloan J., Gray J.E. (2018). Stomatal Development: Focusing on the Grasses. Curr. Opin. Plant Biol..

[18] McKown K.H., Bergmann D.C. (2018). Grass Stomata. Curr. Biol..

[19] Nunes T.D.G., Zhang D., Raissig M.T. (2020). Form, Development and Function of Grass Stomata. Plant J..

[20] Merilo E., Jõesaar I., Brosché M., Kollist H. (2014). To Open or to Close: Species-Specific Stomatal Responses to Simultaneously Applied Opposing Environmental Factors. New Phytol..

[21] McAusland L., Vialet-Chabrand S., Davey P., Baker N.R., Brendel O., Lawson T. (2016). Effects of Kinetics of Light-Induced Stomatal Responses on Photosynthesis and Water-Use Efficiency. New Phytol..

[22] Haworth M., Scutt C.P., Douthe C., Marino G., Gomes M.T.G., Loreto F., Flexas J., Centritto M. (2018). Allocation of the Epidermis to Stomata Relates to Stomatal Physiological Control: Stomatal Factors Involved in the Evolutionary Diversification of the Angiosperms and Development of Amphistomaty. Environ. Exp. Bot..

[23] Franks P.J., Farquhar G.D. (2007). The Mechanical Diversity of Stomata and its Significance in Gas-Exchange Control. Plant Physiol..

[24] Schäfer N., Maierhofer T., Herrmann J., Jørgensen M.E., Lind C., von Meyer K., Lautner S., Fromm J., Felder M., Hetherington A.M. (2018). A Tandem Amino Acid Residue Motif in Guard Cell Slac1 Anion Channel of Grasses Allows for the Control of Stomatal Aperture by Nitrate. Curr. Biol..

[25] Franks P.J., Beerling D.J. (2009). Maximum Leaf Conductance Driven by CO_2_ Effects on Stomatal Size and Density over Geologic Time. PNAS.

[26] Takahashi S., Badger M.R. (2011). Photoprotection in Plants: A New Light on Photosystem II Damage. Trends Plant Sci..

[27] Marchin R.M., Turnbull T.L., Deheinzelin A.I., Adams M.A. (2017). Does Triacylglycerol (TAG) Serve a Photoprotective Function in Plant Leaves? An Examination of Leaf Lipids under Shading and Drought. Physiol. Plant..

[28] Gołębiowska-Pikania G., Kopeć P., Surówka E., Janowiak F., Krzewska M., Dubas E., Nowicka A., Kasprzyk J., Ostrowska A., Malaga S. (2017). Changes in Protein Abundance and Activity Induced by Drought during Generative Development of Winter Barley (*Hordeum vulgare* L.). J. Proteomics.

[29] Lee H.G., Park M.E., Park B.Y., Kim H.U., Seo P.J. (2019). The *Arabidopsis* MYB96 Transcription Factor Mediates Aba-Dependent Triacylglycerol Accumulation in Vegetative Tissues under Drought Stress Conditions. Plants.

[30] Lu J., Xu Y., Wang J., Singer S.D., Chen G. (2020). The Role of Triacylglycerol in Plant Stress Response. Plants.

[31] Mueller S.P., Unger M., Guender L., Fekete A., Mueller M.J. (2017). Diacylglycerol Acyltransferase-Mediated Triacylglyerol Synthesis Augments Basal Thermotolerance. Plant Physiol..

[32] Perlikowski D., Kierszniowska S., Sawikowska A., Krajewski P., Rapacz M., Eckhardt Ä., Kosmala A. (2016). Remodeling of Leaf Cellular Glycerolipid Composition under Drought and Re-Hydration Conditions in Grasses from the *Lolium-Festuca* Complex. Front. Plant Sci..

[33] Perlikowski D., Augustyniak A., Skirycz A., Pawłowicz I., Masajada K., Michaelis Ä., Kosmala A. (2020). Efficient Root Metabolism Improves Drought Resistance of *Festuca arundinacea*. Plant Cell Physiol..

[34] Fan J., Yu L., Xu C. (2017). A central role for Triacylglycerol in Membrane Lipid Breakdown, Fatty Acid β-Oxidation, and Plant Survival under Extended Darkness. Plant Physiol..

[35] Yang Y., Benning C. (2018). Functions of Triacylglycerols during Plant Development and Stress. Curr. Opin. Biotechnol..

[36] Humphreys M.W., Canter P.J., Thomas H.M. (2003). Advances in Introgression Technologies for Precision Breeding within the *Lolium—Festuca* Complex. Ann. Appl. Biol..

[37] Wang S., Li H., Lin C. (2013). Physiological, Biochemical and Growth Responses of Italian Ryegrass to Butachlor Exposure. Pestic. Biochem. Physiol..

[38] Cyriac D., Hofmann R.W., Stewart A., Sathish P., Winefield C.S., Moot D.J. (2018). Intraspecific Differences in Long-Term Drought Tolerance in Perennial Ryegrass. PLoS ONE.

[39] Li M., Sheng G., Wu Y., Yu Z., Bañuelos G.S., Yu H. (2014). Enhancement of Nitrogen and Phosphorus Removal from Eutrophic Water by Economic Plant Annual Ryegrass (*Lolium multiflorum*) with Ion Implantation. Environ. Sci. Pollut. Res..

[40] Perlikowski D., Augustyniak A., Masajada K., Skirycz A., Soja A.M., Michaelis Ä., Wolter G., Kosmala A. (2019). Structural and Metabolic Alterations in Root Systems under Limited Water Conditions in Forage Grasses of *Lolium-Festuca* Complex. Plant Sci..

[41] Franca A., Loi A., Davies W. (1998). Selection of Annual Ryegrass for Adaptation to Semi-Arid Conditions. Eur. J. Agron..

[42] Humphreys M.W., Thomas H. (1993). Improved Drought Resistance in Introgression Lines Derived from *Lolium multiflorum* x *Festuca arundinacea* Hybrids. Plant Breed..

[43] Kemesyte V., Statkeviciute G., Brazauskas G. (2017). Perennial Ryegrass Yield Performance under Abiotic Stress. Crop Sci..

[44] Kosmala A., Perlikowski D., Pawłowicz I., Rapacz M. (2012). Changes in the Chloroplast Proteome Following Water Deficit and Subsequent Watering in a High- and a Low-Drought-Tolerant Genotype of *Festuca arundinacea*. J. Exp. Bot..

[45] Lechowicz K., Pawłowicz I., Perlikowski D., Arasimowicz-Jelonek M., Majka J., Augustyniak A., Rapacz M., Kosmala A. (2020). Two *Festuca* species—*F. arundinacea* and *F. glaucescens*—Differ in the Molecular Response to Drought, while their Physiological Response is Similar. Int. J. Mol. Sci..

[46] Perlikowski D., Kosmala A., Rapacz M., Kościelniak J., Pawlowicz I., Zwierzykowski Z. (2014). Influence of Short-Term Drought Conditions and Subsequent Re-Watering on the Physiology and Proteome of *Lolium multiflorum/Festuca arundinacea* Introgression Forms, with Contrasting Levels of Tolerance to Long-Term Drought. Plant Biol..

[47] Perlikowski D., Czyżniejewski M., Marczak Ł., Augustyniak A., Kosmala A. (2016). Water Deficit Affects Primary Metabolism Differently in Two *Lolium multiflorum/Festuca arundinacea* Introgression Forms with a Distinct Capacity for Photosynthesis and Membrane Regeneration. Front. Plant Sci..

[48] Kosmala A., Zwierzykowski Z., Zwierzykowska E., Łuczak M., Rapacz M., Gasior D., Humphreys M. (2007). Introgression Mapping of Genes For Winter Hardiness and Frost Tolerance Transferred from *Festuca arundinacea* into *Lolium multiflorum*. J. Hered..

[49] Augustyniak A., Perlikowski D., Rapacz M., Kościelniak J., Kosmala A. (2018). Insight into Cellular Proteome of *Lolium multiflorum/Festuca arundinacea* Introgression Forms to Decipher Crucial Mechanisms of Cold Acclimation in Forage Grasses. Plant Sci..

[50] Płażek A., Pociecha E., Augustyniak A., Masajada K., Dziurka M., Majka J., Perlikowski D., Pawłowicz I., Kosmala A. (2018). Dissection of Resistance to *Microdochium nivale* in *Lolium multiflorum/Festuca arundinacea* Introgression Forms. Plant Physiol. Biochem..

[51] Cheng L., Wang Y., He Q., Li H., Zhang X., Zhang F. (2016). Comparative Proteomics Illustrates the Complexity of Drought Resistance Mechanisms in Two Wheat (*Triticum aestivum* L.) Cultivars under Dehydration and Rehydration. BMC Plant Biol..

[52] Pan L., Meng C., Wang J., Ma X., Fan X., Yang Z., Zhou M., Zhang X. (2018). Integrated Omics Data of Two Annual Ryegrass (*Lolium multiflorum* L.) Genotypes Reveals Core Metabolic Processes under Drought Stress. BMC Plant Biol..

[53] Loggini B., Scartazza A., Brugnoli E., Navari-Izzo F. (1999). Antioxidative Defense System, Pigment Composition, and Photosynthetic Efficiency in Two Wheat Cultivars Subjected to Drought. Plant Physiol..

[54] Bian S., Jiang Y. (2009). Reactive Oxygen Species, Antioxidant Enzyme Activities and Gene Expression Patterns in Leaves and Roots of Kentucky Bluegrass in Response to Drought Stress and Recovery. Sci. Hortic. (Amsterdam).

[55] Shao H.B., Liang Z.S., Shao M.A., Wang B.C. (2005). Changes of Anti-Oxidative Enzymes and Membrane Peroxidation for Soil Water Deficits among 10 Wheat Genotypes at Seedling Stage. Colloids Surf. B Biointerfaces.

[56] Ahmed I.M., Nadira U.A., Bibi N., Cao F., He X., Zhang G., Wu F. (2015). Secondary Metabolism and Antioxidants are Involved in the Tolerance to Drought and Salinity, Separately and Combined, in Tibetan Wild Barley. Environ. Exp. Bot..

[57] Beechey-Gradwell Z., Cooney L., Winichayakul S., Andrews M., Hea S.Y., Crowther T., Roberts N. (2020). Storing Carbon in Leaf Lipid Sinks Enhances Perennial Ryegrass Carbon Capture Especially under High N and Elevated CO_2_. J. Exp. Bot..

[58] Paul M.J., Eastmond P.J. (2020). Turning Sugar into Oil: Making Photosynthesis Blind to Feedback Inhibition. J. Exp. Bot..

[59] Doheny-Adams T., Hunt L., Franks P.J., Beerling D.J., Gray J.E. (2012). Genetic Manipulation of Stomatal Density Influences Stomatal Size, Plant Growth and Tolerance to Restricted Water Supply across a Growth Carbon Dioxide Gradient. Philos. Trans. R. Soc. B Biol. Sci..

[60] Hughes J., Hepworth C., Dutton C., Dunn J.A., Hunt L., Stephens J., Waugh R., Cameron D.D., Gray J.E. (2017). Reducing Stomatal Density in Barley Improves Drought Tolerance without Impacting on Yield. Plant Physiol..

[61] Caine R.S., Yin X., Sloan J., Harrison E.L., Mohammed U., Fulton T., Biswal A.K., Dionora J., Chater C.C., Coe R.A. (2019). Rice with Reduced Stomatal Density Conserves Water and Has Improved Drought Tolerance under Future Climate Conditions. New Phytol..

[62] Raissig M.T., Matos J.L., Gil M.X.A., Kornfeld A., Bettadapur A., Abrash E., Allison H.R., Badgley G., Vogel J.P., Berry J.A. (2017). Mobile MUTE Specifies Subsidiary Cells to Build Physiologically Improved Grass Stomata. Science.

[63] Rapacz M., Wójcik-Jagła M., Fiust A., Kalaji H.M., Koscielniak J. (2019). Genome-Wide Associations of Chlorophyll Fluorescence Ojip Transient Parameters Connected with Soil Drought Response in Barley. Front. Plant Sci..

[64] Cheng Z., Dong K., Ge P., Bian Y., Dong L., Deng X., Li X., Yan Y. (2015). Identification of Leaf Proteins Differentially Accumulated between Wheat Cultivars Distinct in Their Levels of Drought Tolerance. PLoS ONE.

[65] Das A., Eldakak M., Paudel B., Kim D.-W., Hemmati H., Basu C., Rohila J.S. (2016). Leaf Proteome Analysis Reveals Prospective Drought and Heat Stress Response Mechanisms in Soybean. Biomed. Res. Int..

[66] Liu D., Li W., Cheng J., Hou L. (2015). *AtPGK2*, a Member of PGKs Gene Family in *Arabidopsis*, has a Positive Role in Salt Stress Tolerance. Plant Cell. Tissue Organ Cult..

[67] Joshi R., Karan R., Singla-Pareek S.L., Pareek A. (2016). Ectopic Expression of Pokkali Phosphoglycerate Kinase-2 (OsPGK2-P) Improves Yield in Tobacco Plants under Salinity Stress. Plant Cell Rep..

[68] Wang X., Du T., Huang J., Peng S., Xiong D. (2018). Leaf Hydraulic Vulnerability Triggers the Decline in Stomatal and Mesophyll Conductance During Drought in Rice. J. Exp. Bot..

[69] Li W., Zhang S., Shan L. (2007). Responsibility of Non-Stomatal Limitations for the Reduction of Photosynthesis-Response of Photosynthesis and Antioxidant Enzyme Characteristics in Alfalfa (*Medicago sativa* L.) Seedlings to Water Stress and Rehydration. Front. Agric. China.

[70] Miller G., Suzuki N., Ciftci-Yilmaz S., Mittler R. (2010). Reactive Oxygen Species Homeostasis and Signalling During Drought and Salinity Stresses. Plant, Cell Environ..

[71] Jiang M., Zhang J. (2002). Water Stress-Induced Abscisic Acid Accumulation Triggers the Increased Generation of Reactive Oxygen Species and Up-Regulates the Activities of Antioxidant Enzymes in Maize Leaves. J. Exp. Bot..

[72] Luna C.M. (2004). Drought Controls on H_2_O_2_ Accumulation, Catalase (CAT) Activity and CAT Gene Expression in Wheat. J. Exp. Bot..

[73] Mittler R. (2017). ROS Are Good. Trends Plant Sci..

[74] Huseynova I.M., Aliyeva D.R., Aliyev J.A. (2014). Subcellular Localization and Responses of Superoxide Dismutase Isoforms in Local Wheat Varieties Subjected to Continuous Soil Drought. Plant Physiol. Biochem..

[75] Leonowicz G., Trzebuniak K.F., Zimak-Piekarczyk P., Ślesak I., Mysliwa-Kurdziel B. (2018). The activity of Superoxide Dismutases (SODs) at the Early Stages of Wheat Deetiolation. PLoS ONE.

[76] Guo Z., Ou W., Lu S., Zhong Q. (2006). Differential Responses of Antioxidative System to Chilling and Drought in Four Rice Cultivars Differing in Sensitivity. Plant Physiol. Biochem..

[77] Rubio M.C., Gonzalez E.M., Minchin F.R., Webb K.J., Arrese-Igor C., Ramos J., Becana M. (2002). Effects of Water Stress on Antioxidant Enzymes of Leaves and Nodules of Transgenic Alfalfa Overexpressing Superoxide Dismutases. Physiol. Plant..

[78] Mittler R., Vanderauwera S., Gollery M., Van Breusegem F. (2004). Reactive Oxygen Gene Network of Plants. Trends Plant Sci..

[79] Laxa M., Liebthal M., Telman W., Chibani K., Dietz K.J. (2019). The Role of the Plant Antioxidant System in Drought Tolerance. Antioxidants.

[80] Fan J., Yan C., Xu C. (2013). Phospholipid: Diacylglycerol Acyltransferase- Mediated Triacylglycerol Biosynthesis is Crucial for Protection against Fatty Acid-Induced Cell Death in Growing Tissues of *Arabidopsis*. Plant J..

[81] Gasulla F., Vom Dorp K., Dombrink I., Zähringer U., Gisch N., Dörmann P., Bartels D. (2013). The Role of Lipid Metabolism in the Acquisition of Desiccation Tolerance in *Craterostigma plantagineum*: A Comparative Approach. Plant J..

[82] Fan J., Yan C., Roston R., Shanklin J., Xu C. (2014). *Arabidopsis* lipins, PDAT1 Acyltransferase, and SDP1 Triacylglycerol Lipase Synergistically Direct Fatty Acids toward Β-Oxidation, Thereby Maintaining Membrane Lipid Homeostasis. Plant Cell.

[83] Chaffai R., Cherif A. (2020). The cadmium-induced changes in the polar and neutral lipid compositions suggest the involvement of triacylglycerol in the defense response in maize. Physiol. Mol. Biol. Plants.

[84] Flexas J. (2016). Genetic Improvement of Leaf Photosynthesis and Intrinsic Water Use Efficiency in C3 Plants: Why So Much Little Success?. Plant Sci..

[85] Schneider C.A., Rasband W.S., Eliceiri K.W. (2012). NIH Image to ImageJ: 25 Years of Image Analysis. Nat. Methods.

[86] Hurkman W.J., Tanaka C.K. (1986). Solubilization of Plant Membrane Proteins for Analysis by Two-Dimensional Gel Electrophoresis. Plant Physiol..

[87] Pawłowicz I., Kosmala A., Rapacz M. (2012). Expression Pattern of the psbO Gene and its Involvement in Acclimation of the Photosynthetic Apparatus during Abiotic Stresses in *Festuca arundinacea* and *F. pratensis*. Acta Physiol. Plant..

[88] Sibley J.A., Lehninger A.L. (1949). Determination of Aldolase in Animal Tissues. J. Biol. Chem..

[89] Willard J.M., Gibbs M. (1968). Role of Aldolase in Photosynthesis. II Demonstration of Aldolase Types in Photosynthetic Organisms. Plant Physiol..

[90] Dhindsa R.S., Plumb-Dhindsa P., Thorpe T.A. (1981). Leaf Senescence: Correlated with Increased Levels of Membrane Permeability and Lipid Peroxidation, and Decreased Levels of Superoxide Dismutase and Catalase. J. Exp. Bot..

[91] Bradford M.M. (1976). A Rapid and Sensitive Method for the Quantitation of Microgram Quantities of Protein Utilizing the Principle of Protein-Dye Binding. Anal. Biochem..

[92] Heath R.L., Packer L. (1968). Photoperoxidation in Isolated Chloroplasts. I. Kinetics and Stoichiometry of Fatty Acid Peroxidation. Arch. Biochem. Biophys..

[93] Doke N. (1983). Involvement of Superoxide Anion Generation in The Hypersensitive Response of Potato Tuber Tissues to Infection with an Incompatible Race of *Phytophthora infestans* and to the Hyphal Wall Components. Physiol. Plant Pathol..

[94] Arasimowicz M., Floryszak-Wieczorek J., Milczarek G., Jelonek T. (2009). Nitric Oxide, Induced by Wounding, Mediates Redox Regulation in Pelargonium Leaves. Plant Biol..

[95] Becana M., Aparicio-Tejo P., Jose Irigoyen J., Sanchez-Diaz M. (1986). Some Enzymes of Hydrogen Peroxide Metabolism in Leaves and Root Nodules of *Medicago sativa*. Plant Physiol..

[96] Arasimowicz-Jelonek M., Floryszak-Wieczorek J., Gzyl J., Chmielowska-Bak J. (2013). Homocysteine Over-Accumulation as the Effect of Potato Leaves Exposure to Biotic Stress. Plant Physiol. Biochem..

[97] Giavalisco P., Li Y., Matthes A., Eckhardt A., Hubberten H.M., Hesse H., Segu S., Hummel J., Köhl K., Willmitzer L. (2011). Elemental Formula Annotation of Polar and Lipophilic Metabolites Using 13C, 15N and 34S Isotope Labelling, in Combination with High-Resolution Mass Spectrometry. Plant J..

[98] Bromke M.A., Hochmuth A., Tohge T., Fernie A.R., Giavalisco P., Burgos A., Willmitzer L., Brotman Y. (2015). Liquid Chromatography High-Resolution Mass Spectrometry for Fatty acid Profiling. Plant J..

