# Supplementary material for: Adjustment of Photosynthetic and Antioxidant Activities to Water Deficit Is Crucial in the Drought Tolerance of Lolium multiflorum/Festuca arundinacea Introgression Forms
Source: Int J Mol Sci. 2020 Aug 6;21(16):5639. doi: 10.3390/ijms21165639 (PMC7460672; doi:10.3390/ijms21165639)
Supplement: Supplementary file 1 [file ijms-21-05639-s001.pdf]

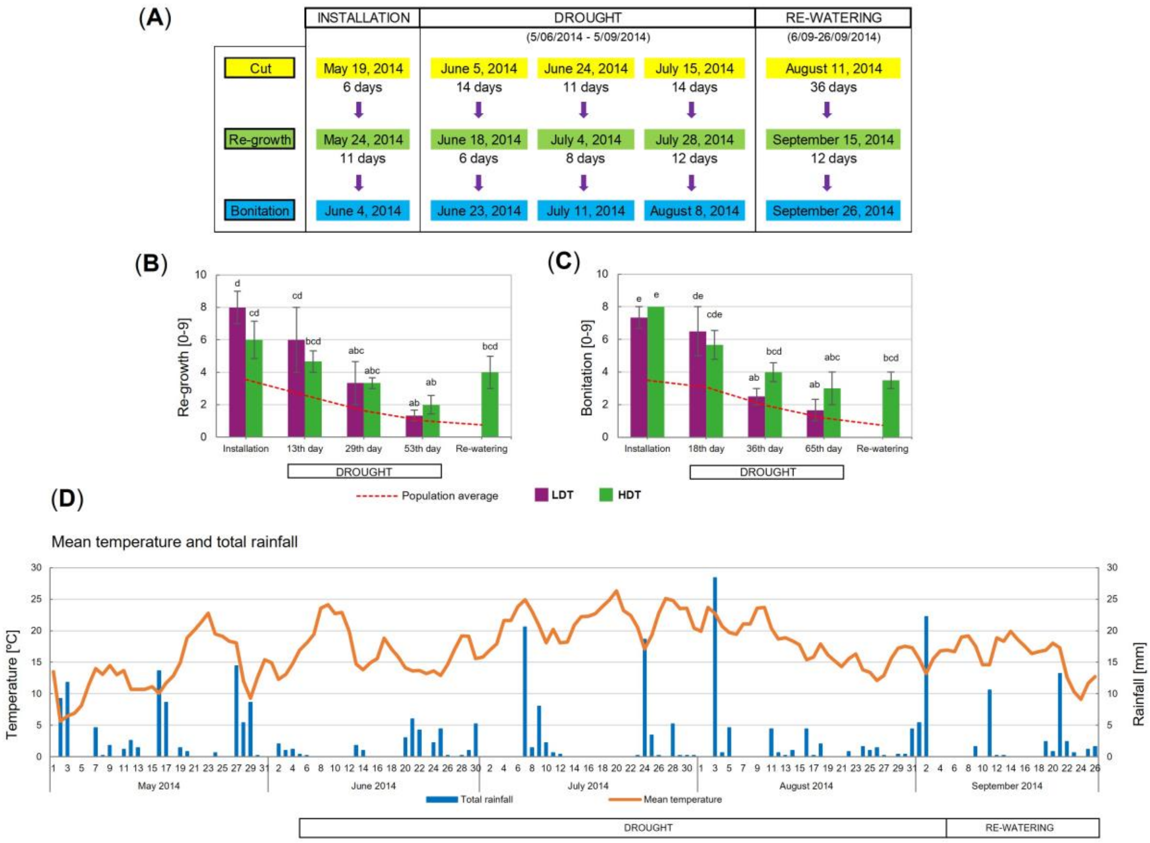

Figure S1. (**A**) Scheme of the field experiment. (**B**) Mean of re-growth (**C**) and bonitation of two *L. multiflorum/F. arundinacea* introgression forms (low- and high-drought tolerant—LDT and HDT, respectively) and mean of these parameters for the population. Error bars represent the standard errors of three biological replicates. Homogeneous groups are denoted by the same letter according to Fisher's LSD test ($p = 0.01$). (**D**) Mean air temperature and total rainfall.

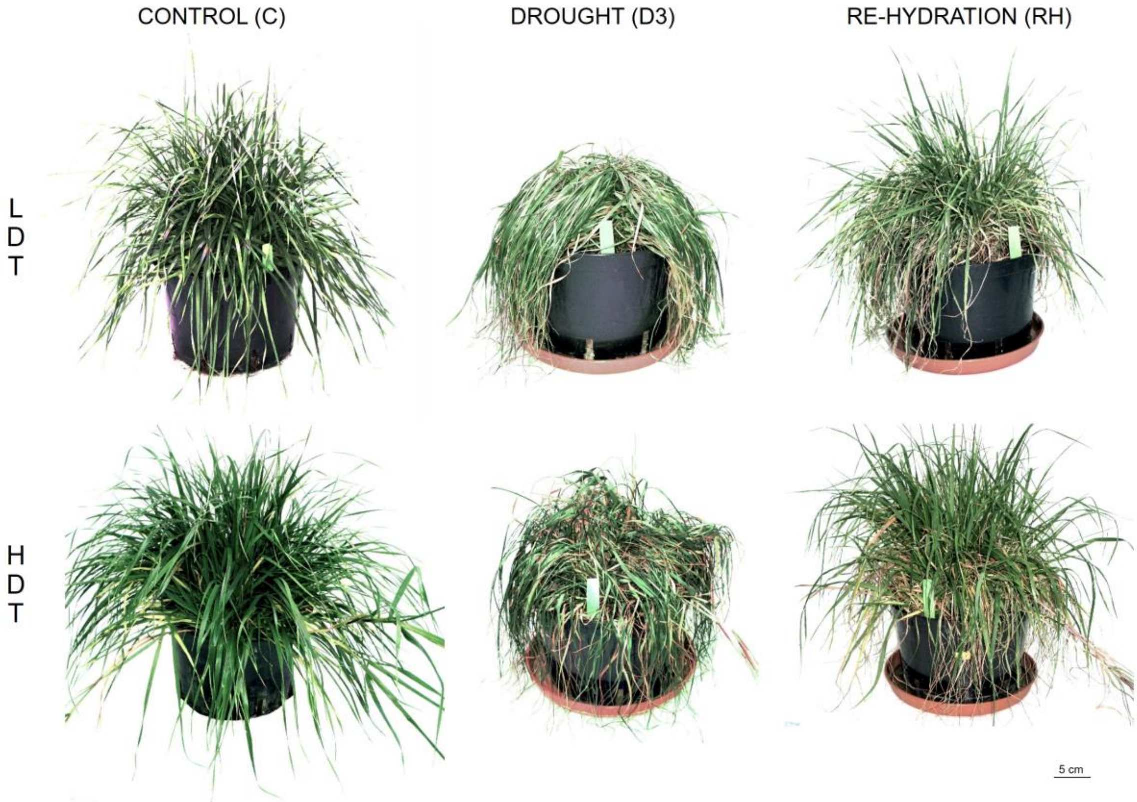

Figure S2. Photographs presenting the phenotypes of the two *L. multiflorum*/*F. arundinacea* introgression forms (low- and high-drought tolerant—LDT and HDT, respectively) under the control conditions (C), on the 10th day of drought (D3) and after re-watering (RH).

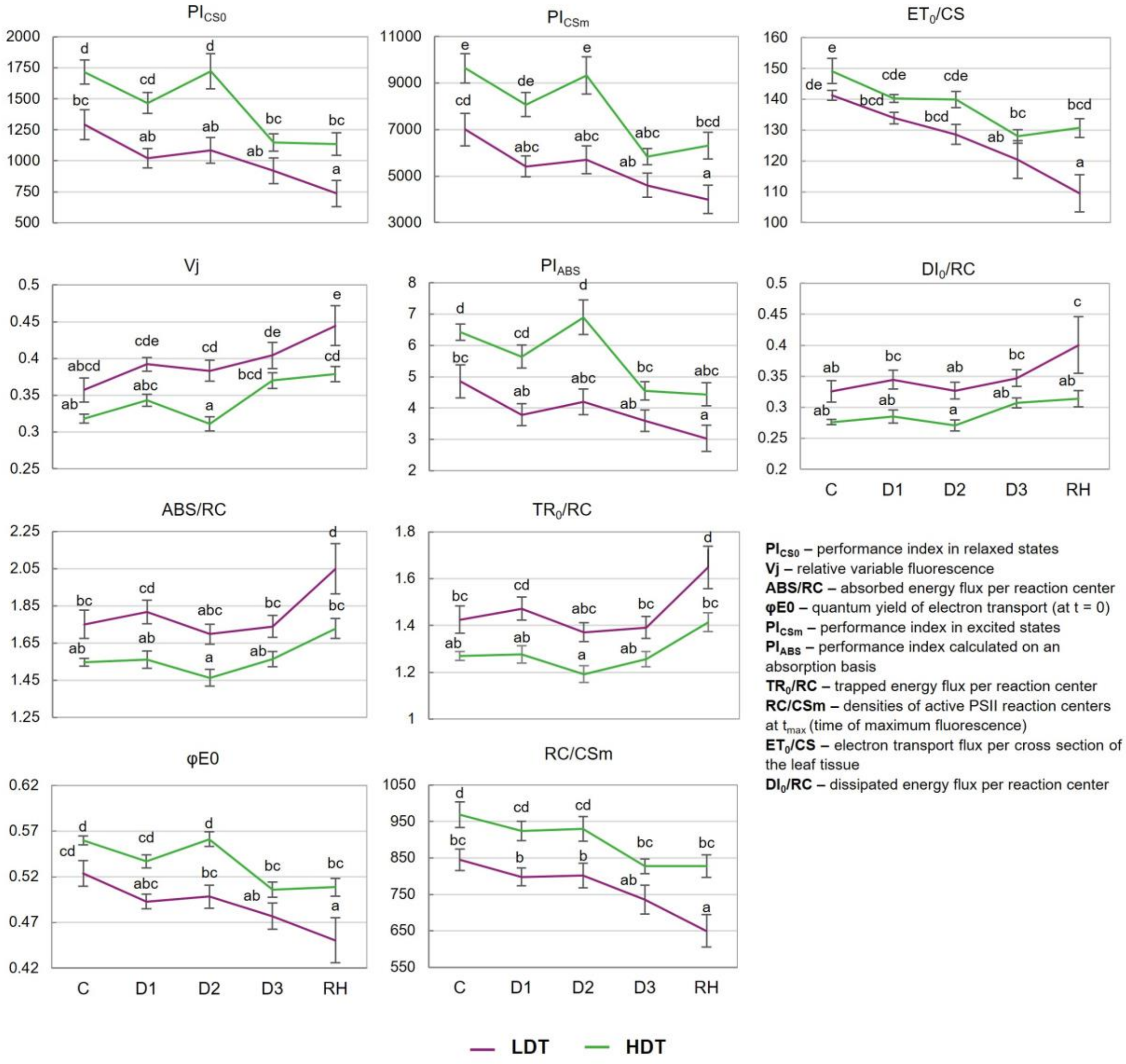

Figure S3. Chlorophyll fluorescence parameters: PI_{CS0}, Vj, ABS/RC, φE0, PI_{CSm}, PI_{ABS}, TR_0/RC, RC/CSm, ET_0/CS and DI_0/RC in two *L. multiflorum*/*F. arundinacea* introgression forms (low- and high-drought tolerant—LDT and HDT, respectively) before stress treatment (C), on the 3rd (D1), 6th (D2), and 10th (D3) day of the water deficit and 10 days after re-hydration (RH). The data represent the means of ten individual measurements; error bars represent the standard errors. Homogeneous groups are denoted by the same letter according to Fisher's LSD test ($p = 0.01$).
